# Integrative analysis of the cancer genome atlas and cancer cell lines encyclopedia large-scale genomic databases: MUC4/MUC16/MUC20 signature is associated with poor survival in human carcinomas

**DOI:** 10.1186/s12967-018-1632-2

**Published:** 2018-09-20

**Authors:** Nicolas Jonckheere, Isabelle Van Seuningen

**Affiliations:** Inserm, CHU Lille, UMR-S 1172-JPARC-Jean-Pierre Aubert Research Center, Team “Mucins, epithelial differentiation and carcinogenesis”, Univ. Lille, 59000 Lille, France

**Keywords:** MUC4, TCGA, CCLE, Patient survival, Biomarker

## Abstract

**Background:**

MUC4 is a membrane-bound mucin that promotes carcinogenetic progression and is often proposed as a promising biomarker for various carcinomas. In this manuscript, we analyzed large scale genomic datasets in order to evaluate *MUC4* expression, identify genes that are correlated with *MUC4* and propose new signatures as a prognostic marker of epithelial cancers.

**Methods:**

Using cBioportal or SurvExpress tools, we studied *MUC4* expression in large-scale genomic public datasets of human cancer (the cancer genome atlas, TCGA) and cancer cell line encyclopedia (CCLE).

**Results:**

We identified 187 co-expressed genes for which the expression is correlated with *MUC4* expression. Gene ontology analysis showed they are notably involved in cell adhesion, cell–cell junctions, glycosylation and cell signaling. In addition, we showed that *MUC4* expression is correlated with *MUC16* and *MUC20*, two other membrane-bound mucins. We showed that MUC4 expression is associated with a poorer overall survival in TCGA cancers with different localizations including pancreatic cancer, bladder cancer, colon cancer, lung adenocarcinoma, lung squamous adenocarcinoma, skin cancer and stomach cancer. We showed that the combination of *MUC4*, *MUC16* and *MUC20* signature is associated with statistically significant reduced overall survival and increased hazard ratio in pancreatic, colon and stomach cancer.

**Conclusions:**

Altogether, this study provides the link between (i) MUC4 expression and clinical outcome in cancer and (ii) MUC4 expression and correlated genes involved in cell adhesion, cell–cell junctions, glycosylation and cell signaling. We propose the MUC4/MUC16/MUC20^high^ signature as a marker of poor prognostic for pancreatic, colon and stomach cancers.

**Electronic supplementary material:**

The online version of this article (10.1186/s12967-018-1632-2) contains supplementary material, which is available to authorized users.

## Background

The cancer genome atlas (TCGA) was developed by National Cancer Institute (NCI) and National Human Genome Research Institute (NHGRI) in order to provide comprehensive mapping of the key genomic changes that occur during carcinogenesis. Datasets of more than 11,000 patients of 33 different types of tumors are publically available. In parallel, cancer cell line encyclopedia (CCLE), a large-scale genomic dataset of human cancer cell lines, was generated by the Broad Institute and Novartis in order to reflect the genomic diversity of human cancers and provide complete preclinical datasets for mutation, copy number variation and mRNA expression studies [1]. In order to analyse this kind of large scale datasets, several useful online tools have been created. cBioportal is an open-access database analysis tool developed at the Memorial Sloan-Kettering Cancer Centre (MSKCC) to analyze large-scale cancer genomics data sets [2, 3]. SurvExpress is another online tool for biomarker validation using 225 datasets available and therefore provide key information linking gene expression and the impact on cancer outcome [4].

Mucins are large high molecular weight glycoproteins that are classified in two sub groups: (i) the secreted mucins that are responsible of rheologic properties of mucus and (ii) the membrane-bound mucins that include MUC4, MUC16 and MUC20 [5, 6]. *MUC4* was first discovered in our laboratory 25 years ago from a tracheobronchial cDNA library [7]. MUC4 is characterized by a long hyper-glycosylated extracellular domain, Epidermal Growth Factor (EGF)-like domains, a hydrophobic transmembrane domain, and a short cytoplasmic tail. MUC4 also contains NIDO, AMOP and vWF-D domains [8]. A direct interaction between MUC4 and its membrane partner, the oncogenic receptor ErbB2, alters downstream signaling pathways [9]. MUC4 is expressed at the surface of epithelial cells from gastrointestinal and respiratory tracts [10] and has been studied in various cancers where it is generally overexpressed and described as an oncomucin and has been proposed as an attractive prognostic tumor biomarker. Its biological role has been mainly evaluated in pancreatic, ovarian, esophagus and lung cancers [9, 11–14]. Other membrane-bound mucins MUC16 and MUC20 share some functional features but evolved from distinct ancestors [15]. *MUC20* gene is located on the chromosomic region 3q29 close to *MUC4*. MUC16, also known as the CA125 antigen, is a routinely used serum marker for the diagnosis of ovarian cancer [16]. Both mucins favor tumor aggressiveness and are associated with poor overall survival and could be proposed as prognosis factors [16–18].

In this manuscript, we have used the online tools cBioportal, DAVID6.8 and SurvExpress in order to (i) evaluate *MUC4* expression in various carcinomas, (ii) identify genes that are correlated with *MUC4* and evaluate their roles and (iii) propose *MUC4*/*MUC16*/*MUC20* combination as a prognostic marker of pancreatic, colon and stomach cancers.

## Methods

### Expression analysis from public datasets

*MUC4* z-score expressions were extracted from databases available at cBioPortal for Cancer Genomics [2, 3]. This portal stores expression data and clinical attributes. The z-score for *MUC4* mRNA expression is determined for each sample by comparing mRNA expression to the distribution in a reference population harboring typical expression for the gene. The query “MUC4” was realized in CCLE (881 samples, Broad Institute, Novartis Institutes for Biomedical Research) [1] and in all TCGA datasets available (13,489 human samples, TCGA Research Network (http://cancergenome.nih.gov/)). The mRNA expression from selected data was plotted in relation to the clinical attribute (tumor type and histology) in each sample. MUC4 expression was analyzed in normal tissues by using the Genome Tissue Expression (GTEX) tool [19, 20]. Data were extracted from GTEX portal on 06/29/17 (dbGaP accession phs000424.v6.p1) using the 4585 Entrez gene ID.

### DAVID6.8 identification and gene ontology of genes correlated with *MUC4*

We established a list of 187 genes that are correlated with *MUC4* expression in CCLE dataset out of 16208 genes analyzed with cBioportal tool on co-expression tab. These genes harbor a correlation with both Pearson’s and Spearman’s higher than 0.3 or lower than − 0.3. Functional annotation and ontology clustering of the complete list of genes were performed using David Functional Annotation Tool (https://david.ncifcrf.gov/) and Homo sapiens background [21, 22]. Enrichment scores of ontology clusters are provided by the online tool.

Interaction of proteins correlated with MUC4 was determined using String 10 tool (https://string-db.org/) [23]. Edges represent protein–protein associations such as known interactions (from curated databases or experimentally determined), predicted interactions (from gene neighborhood, gene fusion or co-occurrence), text-mining, co-expression or protein homology. The network was divided in 3 clusters based on k-means clustering.

### Methylation and copy number analysis

Using (https://portals.broadinstitute.org/ccle), we extracted mRNA expression of *MUC4*, methylation score (Reduced Representation Bisulfite Sequencing: RRBS) and copy number variations of the genes of interest. The mRNA expression of *MUC4* was plotted in relation to log2 copy number or RRBS score.

### SurvExpress survival analysis

Survival analysis was performed using the SurvExpress online tool available in bioinformatica.mty.itesm.mx/SurvExpress (Aguire Gamboa PLos One 2013). We used the optimized algorithm that generates risk group by sorting prognostic index (higher value of MUC4 for higher risk) and split the two cohorts where the p-value is minimal. Hazard ratio [95% confidence interval (CI)] was also evaluated. The tool also provided a box plot of genes expression and the corresponding p value testing the differences.

### Gene Expression Omnibus microarray

GSE28735 and GSE16515 pancreatic cancer microarrays were analysed from the NCBI Gene Expression Omnibus (GEO) database (http://www.ncbi.nml.nih.gov/geo/). GSE28735 is a dataset containing 45 normal pancreas (adjacent non tumoral, ANT) and 45 tumor (T) tissues from pancreatic ductal adenocarcinoma (PDAC) cases. GSE16515 contains 52 samples (16 had both tumor and normal expression data, and 20 only had tumor data. Data were analysed using GEO2R software. The dataset GSE28735 used Affymetrix GeneChip Human Gene 1.0 ST array. The dataset GSE16515 used the Affymetrix Human Genome U133 Plus 2.0 Array. GSE13507 contains 165 bladder cancer and 58 ANT samples. GSE30219 contains 14 normal lung, 85 adenocarcinomas and 61 squamous cancer samples. GSE40967 contains 566 colorectal cancers and 19 normal mucosae. GSE27342 contains 80 tumors and 80 paired ANT tissues. GSE4587 contains 2 normal, 2 melanomas and 2 metastatic melanomas. GSE14407 contains 12 ovarian adenocarcinomas and 12 normal ovary samples.

### Statistical analysis

For *MUC4* expression analysis, paired and unpaired t test statistical analyses were performed using the Graphpad Prism 6.0 software (Graphpad softwares Inc., La Jolla, CA, USA). p < 0.05 was considered as statistically significant. Receiving operator characteristic (ROC) curves and areas under ROC (AUROC) were evaluated by comparing tumor and ANT values. cBioportal provided Pearson and Spearman tests were performed to analyze correlation of other genes, RRBS score and log2 copy number with* MUC4* expression. DAVID tool provided p value of each ontology enrichment score. SurvExpress tool provided statistical analysis of hazard ratio and overall survival. A Log rank testing evaluated the equality of survival curves between the high and low risk groups.

## Results

### MUC4 expression analysis in databases

**Fig. 1:**
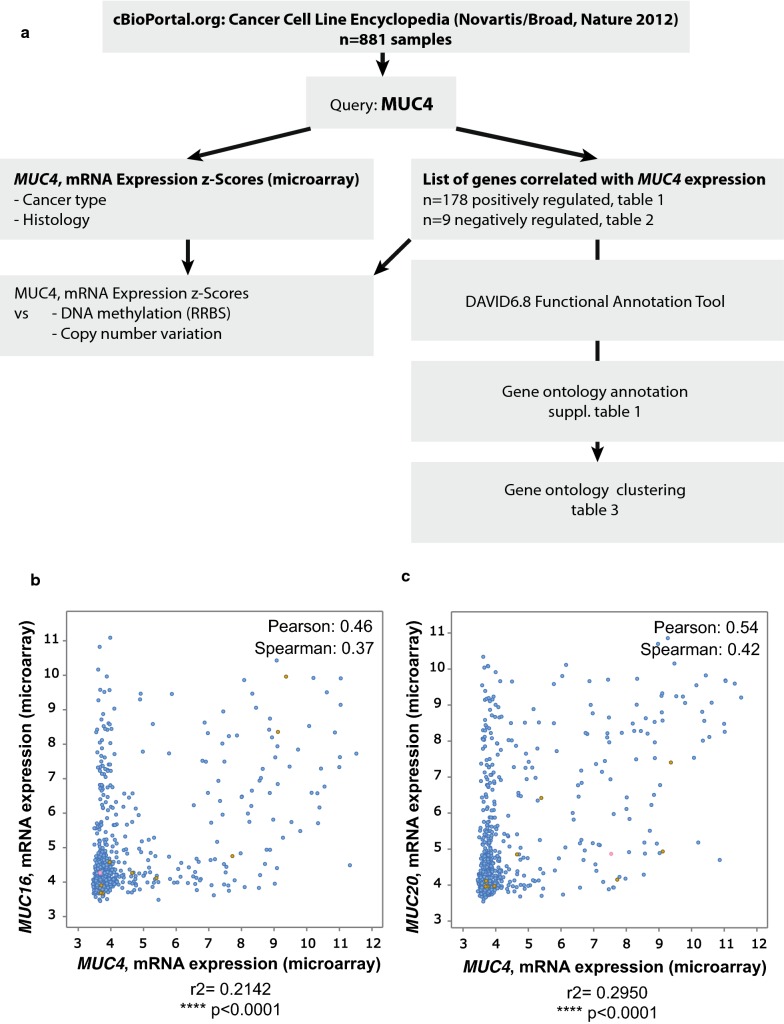
Strategy of analysis of genes correlated with *MUC4* expression in Cancer Cell Line Encyclopedia. **a** Flowchart of MUC4 analysis. *MUC4* mRNA expression z-scores were extracted from Cancer Cell Line Encyclopedia using cBioportal. The list of gene correlated with *MUC4* expression was determined by using the co-expression tool. Genes presenting a Pearson’s correlation higher than 0.3 or lower than − 0.3 were selected. Spearman analysis was performed subsequently. Gene ontology annotation and clustering were performed using DAVID 6.8 functional annotation tool. **b** Example of *MUC4*-*MUC16* correlation of mRNA expression. **c** Example of *MUC4*-*MUC20* correlation of mRNA expression

**Fig. 2:**
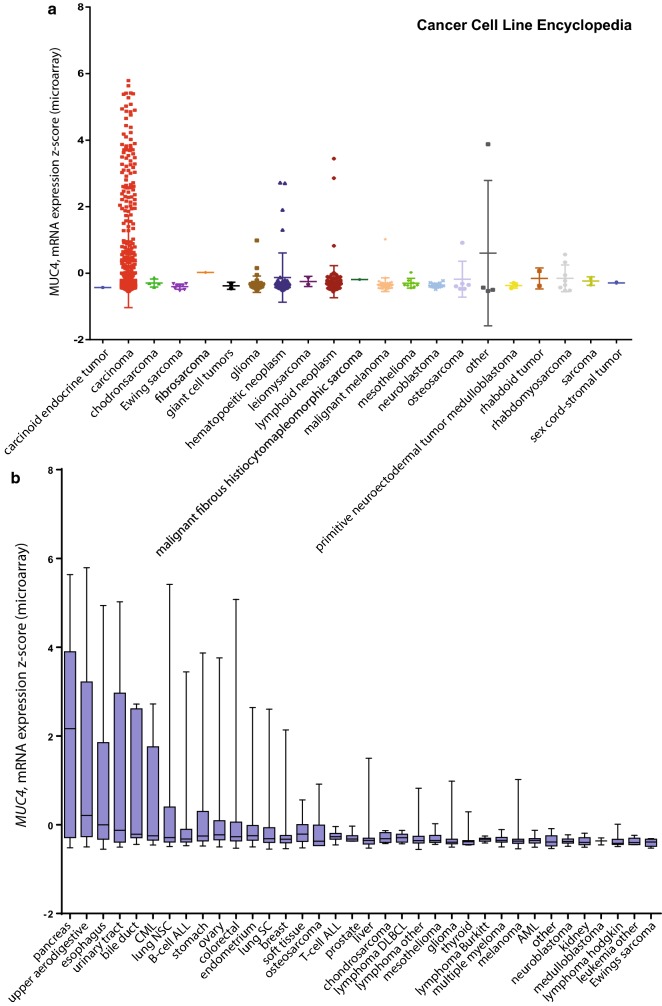
*MUC4* expression in Cancer Cell Line Encyclopedia. *MUC4* mRNA expression z-scores were extracted from Cancer Cell Line Encyclopedia (Novartis/Barretina Nature 2012) using cBioportal. N = 881 samples. Expression data were sorted depending on tumor type (**a**) and histology (**b**)

**Fig. 3:**
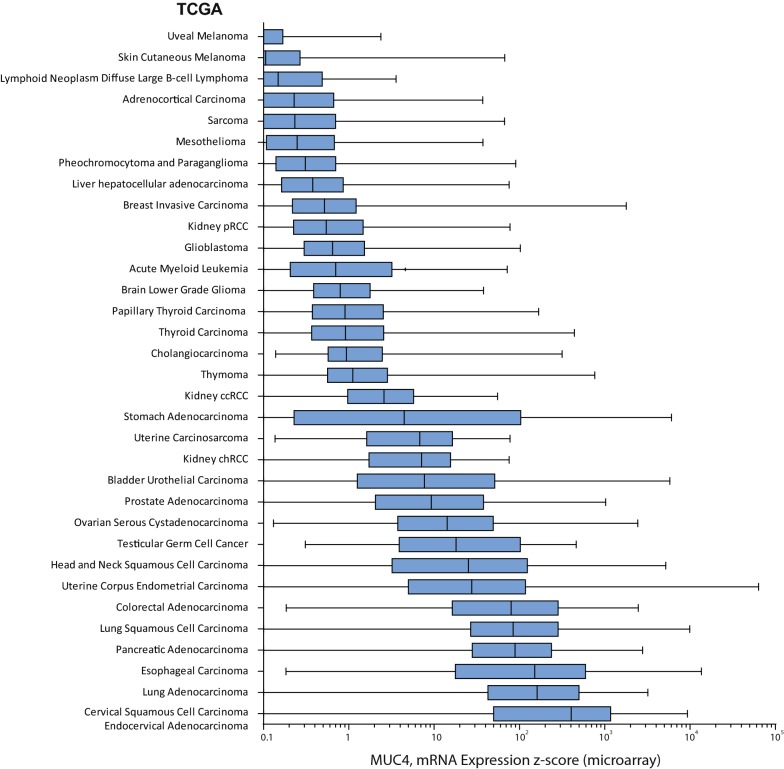
*MUC4* expression in cancer samples from TCGA. *MUC4* mRNA expression z-scores were extracted from TCGA samples using cBioportal. N = 13 489 samples. Expression data were sorted depending on organs

*MUC4* expression was analyzed from databases available at cBioPortal for Cancer Genomics [2, 3]. We queried for *MUC4* mRNA expression in the 881 samples from CCLE [1] (Fig. 1). The oncoprint showed that *MUC4* was altered in 195 samples out of 881 (22%). 188 were amplification (n = 120) or mRNA upregulation (n = 88) (Additional file 1: Figure S1). Results were sorted depending on the tumor type. We mainly observed an important z-score expression of *MUC4* in carcinoma samples (n = 538 samples, p = 0.001) (Fig. 2a). *MUC4* Expression scores were subsequently sorted depending on the organ (Fig. 2b). As expected, pancreatic cancer cell lines harbor the highest *MUC4* expression (n = 35, z-score = 2.166, p = 0.0006 against theoretical control median = 0). Other cell lines from different tissues (lung NSC, esophagus, bile duct, stomach, upper digestive, colorectal, ovary, and urinary tract) showed statistically significant alteration. We also performed a similar analysis on 13 489 human samples retrieved from TCGA by using the cBioportal platform. An important *MUC4* expression z-score was observed in bladder urothelial carcinoma, cervical squamous cell carcinoma/endocervical adenocarcinoma, colorectal carcinoma, esophageal carcinoma, head and neck squamous cell carcinoma, lung adenocarcinoma, lung squamous cell carcinoma, ovarian serous cystadenocarcinoma, pancreatic adenocarcinoma, prostate adenocarcinoma, stomach adenocarcinoma and uterine corpus endometrial carcinoma (Fig. 3). Expression of *MUC4* in normal tissues was analyzed using the GTEX project tool, *MUC4* was expressed in lung, testis, small intestine, terminal, ileum, prostate, vagina, minor salivary gland and esophagus mucosa and transverse colon (Additional file 2: Figure S2). Altogether, this shows that *MUC4* high expression is observed in carcinoma and notably in pancreatic cancer.

### *MUC4* co-regulated genes

Using the co-expression tool on expression data extracted from the 881 samples of CCLE [1], we obtained a list of genes that are co-expressed with *MUC4*. Genes that harbor a correlation with both Pearson’s and Spearman’s higher than 0.3 or lower than − 0.3 were selected. 187 genes are positively (n = 178) or negatively (n = 9) correlated with *MUC4* expression. The better correlated genes were Adhesion G Protein-Coupled Receptor F1 (*ADGRF1*, Pearson’s correlation = 0.56) and Lipocalin2 (*LCN2*, Pearson’s correlation = 0.54) (Table 1). We also observed that expression of other membrane-bound mucins *MUC16* and *MUC20* are positively correlated with *MUC4*. Correlation between *MUC16* and *MUC20* was also observed (not shown). Only few genes were negatively correlated such as *ZEB1* transcription factor or *ST3 Beta*-*Galactoside Alpha*-*2,3*-*Sialyltransferase 2* (*ST3GAL2*) (Table 2).

**Table 1:** List of mRNA positively correlated with MUC4

Correlated gene	Cytoband	Pearson’s correlation	Spearman’s correlation
ADGRF1	6p12.3	0.56	0.40
LCN2	9q34	0.54	0.41
MUC20	3q29	0.54	0.42
C1ORF116	1q32.1	0.52	0.47
SCEL	13q22	0.52	0.43
STEAP4	7q21.12	0.51	0.35
WFDC2	20q13.12	0.48	0.31
GJB3	1p34	0.48	0.35
SH2D3A	19p13.3	0.48	0.45
RNF39	6p21.3	0.47	0.35
PRSS22	16p13.3	0.47	0.41
HS3ST1	4p16	0.46	0.35
GPR87	3q24	0.46	0.35
TACSTD2	1p32	0.46	0.41
MUC16	19p13.2	0.46	0.37
FAM83A	8q24.13	0.45	0.34
LAMC2	1q25-q31	0.45	0.32
B3GNT3	19p13.1	0.45	0.40
CLDN7	17p13.1	0.45	0.44
ELF3	1q32.2	0.44	0.44
MIR205HG	1q32.2	0.44	0.37
PPL	16p13.3	0.44	0.40
MPZL2	11q24	0.44	0.43
TMPRSS4	11q23.3	0.44	0.46
C6ORF132	6p21.1	0.43	0.36
FGFBP1	4p15.32	0.43	0.38
IRF6	1q32.3-q41	0.43	0.44
LAMB3	1q32	0.43	0.31
CDH3	16q22.1	0.43	0.41
SPINT1	15q15.1	0.43	0.42
EHF	11p12	0.43	0.41
CYSRT1	9q34.3	0.42	0.33
MACC1	7p21.1	0.42	0.38
MST1R	3p21.3	0.42	0.41
SERPINB5	18q21.33	0.42	0.39
TMEM30B	14q23.1	0.42	0.40
CLDN4	7q11.23	0.41	0.37
LIPH	3q27	0.41	0.36
ALS2CL	3p21.31	0.41	0.37
ITGB6	2q24.2	0.41	0.37
RAB25	1q22	0.41	0.41
CNKSR1	1p36.11	0.41	0.43
TSPAN1	1p34.1	0.41	0.36
CEACAM6	19q13.2	0.41	0.37
KLK10	19q13	0.41	0.37
UCA1	19p13.12	0.41	0.32
CXCL16	17p13	0.41	0.35
ELMO3	16q22.1	0.41	0.44
PRSS8	16p11.2	0.41	0.42
ST14	11q24-q25	0.41	0.40
TRIM29	11q23.3	0.41	0.37
GRHL2	8q22.3	0.40	0.40
PTK6	20q13.3	0.40	0.34
FLJ23867	1q25.2	0.40	0.31
TMC4	19q13.42	0.40	0.38
CDH1	16q22.1	0.40	0.39
SDR16C5	8q12.1	0.39	0.35
S100A14	1q21.3	0.39	0.38
GJB5	1p35.1	0.39	0.33
JUP	17q21	0.39	0.40
TMC5	16p12.3	0.39	0.42
SCGB1A1	11q12.3	0.39	0.34
MROH6	8q24.3	0.38	0.39
MAL2	8q23	0.38	0.41
ESRP1	8q22.1	0.38	0.42
GALNT3	2q24-q31	0.38	0.38
CBLC	19q13.2	0.38	0.40
FUT3	19p13.3	0.38	0.42
PKP3	11p15	0.38	0.39
EPHA1	7q34	0.37	0.39
AGR2	7p21.3	0.37	0.33
CDS1	4q21.23	0.37	0.37
S100P	4p16	0.37	0.36
ARL14	3q25.33	0.37	0.33
KRTCAP3	2p23.3	0.37	0.41
BIK	22q13.31	0.37	0.38
SFN	1p36.11	0.37	0.41
TMEM125	1p34.2	0.37	0.44
C19ORF33	19q13.2	0.37	0.35
LSR	19q13.12	0.37	0.41
MISP	19p13.3	0.37	0.39
ESRP2	16q22.1	0.37	0.39
PAK6	15q14	0.37	0.37
KRT4	12q13.13	0.37	0.32
ANKRD22	10q23.31	0.37	0.40
MARVELD2	5q13.2	0.36	0.38
LAD1	1q25.1-q32.3	0.36	0.38
F11R	1q21.2-q21.3	0.36	0.44
CGN	1q21	0.36	0.42
ARHGEF16	1p36.3	0.36	0.43
KIAA1522	1p35.1	0.36	0.33
DMKN	19q13.12	0.36	0.34
STAP2	19p13.3	0.36	0.34
EVPL	17q25.1	0.36	0.38
ITGB4	17q25	0.36	0.36
MARVELD3	16q22.2	0.36	0.42
CCDC64B	16p13.3	0.36	0.38
KLF5	13q22.1	0.36	0.35
KRT6A	12q13.13	0.36	0.33
EXPH5	11q22.3	0.36	0.37
PLEKHA7	11p15.1	0.36	0.33
PRRG4	11p13	0.36	0.33
ADAP1	7p22.3	0.35	0.35
IL1RN	2q14.2	0.35	0.36
EPCAM	2p21	0.35	0.38
PVRL4	1q23.3	0.35	0.31
EPS8L1	19q13.42	0.35	0.39
PRRG2	19q13.33	0.35	0.43
FXYD3	19q13.12	0.35	0.37
CRB3	19p13.3	0.35	0.40
MYO5C	15q21	0.35	0.37
TC2 N	14q32.12	0.35	0.38
PLEKHG3	14q23.3	0.35	0.35
FAM83H	8q24.3	0.34	0.39
FRK	6q21-q22.3	0.34	0.31
FAM110C	2p25.3	0.34	0.35
KDF1	1p36.11	0.34	0.40
KLK6	19q13.3	0.34	0.38
SPINT2	19q13.1	0.34	0.39
TTC9	14q24.2	0.34	0.32
FOXA1	14q21.1	0.34	0.36
TJP2	9q13-q21	0.33	0.31
ARHGEF5	7q35	0.33	0.33
MAPK13	6p21.31	0.33	0.32
ZNF165	6p21.3	0.33	0.41
ANXA3	4q21.21	0.33	0.30
B3GNT5	3q28	0.33	0.32
ZBED2	3q13.2	0.33	0.31
GRHL1	2p25.1	0.33	0.38
FERMT1	20p12.3	0.33	0.31
SPRR1A	1q21-q22	0.33	0.31
S100A9	1q21	0.33	0.33
PCSK9	1p32.3	0.33	0.34
CEACAM5	19q13.1-q13.2	0.33	0.33
KLK8	19q13	0.33	0.36
GNA15	19p13.3	0.33	0.32
KRT19	17q21.2	0.33	0.32
TNS4	17q21.2	0.33	0.41
PLEK2	14q23.3	0.33	0.32
DTX4	11q12.1	0.33	0.31
TSPAN15	10q22.1	0.33	0.34
CHMP4C	8q21.13	0.32	0.38
DAPP1	4q25-q27	0.32	0.32
PROM2	2q11.1	0.32	0.37
AIM1L	1p36.11	0.32	0.42
GRHL3	1p36.11	0.32	0.34
MYH14	19q13.33	0.32	0.41
TJP3	19p13.3	0.32	0.40
DSC2	18q12.1	0.32	0.32
LLGL2	17q25.1	0.32	0.40
IL18	11q23.1	0.32	0.32
OVOL1	11q13	0.32	0.40
CORO2A	9q22.3	0.31	0.34
TMEM184A	7p22.3	0.31	0.40
MAP7	6q23.3	0.31	0.33
IL20RA	6q23.3	0.31	0.37
DDR1	6p21.3	0.31	0.32
FAM83B	6p12.1	0.31	0.37
LAMP3	3q26.3-q27	0.31	0.36
OVOL2	20p11.23	0.31	0.41
KCNK1	1q42-q43	0.31	0.35
PTAFR	1p35-p34.3	0.31	0.34
FUT2	19q13.3	0.31	0.38
LRG1	19p13.3	0.31	0.32
ST6GALNAC1	17q25.1	0.31	0.43
GRB7	17q12	0.31	0.38
ATP2C2	16q24.1	0.31	0.42
PLA2G10	16p13.1-p12	0.31	0.39
SCNN1A	12p13	0.31	0.40
TMEM45B	11q24.3	0.31	0.38
EZR	6q25.3	0.30	0.31
ARAP2	4p14	0.30	0.31
CDCP1	3p21.31	0.30	0.30
PTPRU	1p35.3	0.30	0.30
KLC3	19q13	0.30	0.36
EPN3	17q21.33	0.30	0.39
ARHGAP27	17q21.31	0.30	0.35
FA2H	16q23	0.30	0.40

Data were retrieved from 881 samples of Cancer Cell Line Encyclopedia (Novartis/Broad, Nature 2012). Correlation analysis was performed using cBioPortal.org online tool. 178 genes presented a Pearson’s correlation higher than 0.3

**Table 2:** List of mRNA negatively correlated with MUC4

Correlated gene	cytoband	Pearson’s correlation	Spearman’s correlation
SLC35B4	7q33	− 0.30	− 0.32
IFFO1	12p13.3	− 0.30	− 0.36
TTC28	22q12.1	− 0.31	− 0.33
VKORC1	16p11.2	− 0.31	− 0.35
DIXDC1	11q23.1	− 0.31	− 0.31
ATP8B2	1q21.3	− 0.32	− 0.33
ST3GAL2	16q22.1	− 0.32	− 0.31
ZEB1	10p11.2	− 0.33	− 0.35
MTFR1L	1p36.11	− 0.34	− 0.35

Data were retrieved from 881 samples of Cancer Cell Line Encyclopedia (Novartis/Broad, Nature 2012). Correlation analysis was performed using cBioPortal.org online tool. 9 genes presented a Pearson’s correlation lower than **− **0.3

**Fig. 4:**
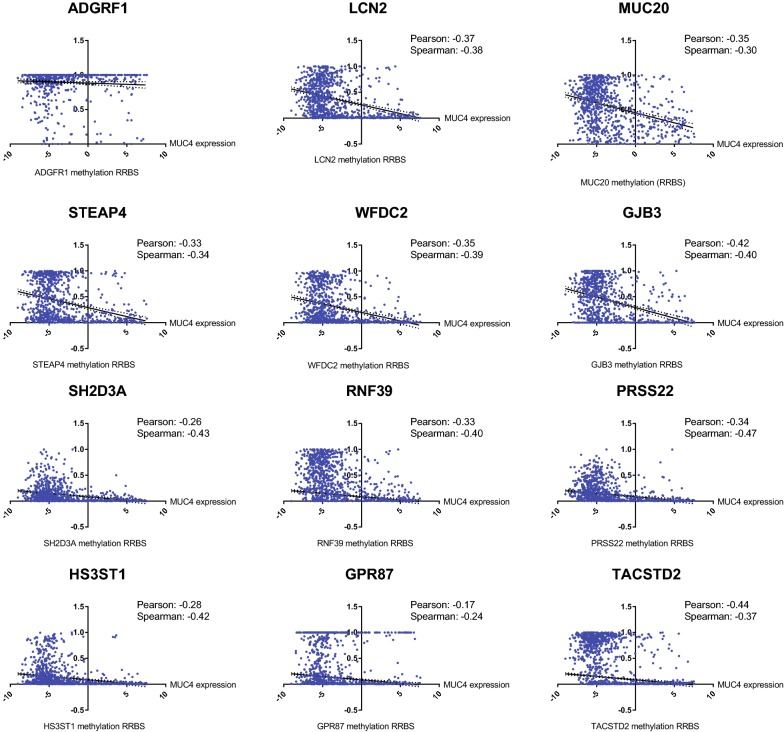
Correlation of *MUC4* expression and methylation of genes correlated with *MUC4*. The top genes were defined as genes harboring Pearson’s correlation higher than 0.5 with *MUC4* expression. *MUC4* mRNA expression and methylation score (Reduced Representation Bisulfite Sequencing: RRBS) of ADGRF1, *LCN2, MUC20, C1ORF116, STEAP4, SCEL, WFDC2, GJB3, SH2D3A, RNF39, PRSS22, HS3ST1, GPR87, TACST2, MUC16, FAM83A, LAMC2, B3GNT3, CLDN7* were extracted using (https://portals.broadinstitute.org/ccle)

Functional Annotation of the complete list of genes and ontology clustering were performed using David Functional Annotation Tool. The gene clustering analysis is presented in Table 3. The complete gene ontologies that are statistically significant are provided in Additional file 3: Table S1. We observed the highest enrichment scores in gene clusters involved in cell adhesion (7.08) and tight junction (5.44) (Table 3). Notably, we observed the correlation of expression of *MUC4* with genes encoding integrins (*ITGB4* and *ITGB6*) and cadherin-type proteins such as *CDH1*, *CDH3*, Desmocollin 2 (*DSC2)*. A strong enrichment of 91 transmembrane proteins was observed including EPH Receptor A1 (*EPHA1*), Epithelial cell adhesion molecule (*EPCAM*), Carcinoembryonic Antigen Related Cell Adhesion Molecule-5 and -6 (*CEACAM5* and *CEACAM6*), C-X-C motif chemokine ligand 16 (*CXCL16*) and ATPase Secretory Pathway Ca^2+^ Transporting 2 (*ATP2C2*). As MUC4 is a glycoprotein, it is interesting to also note the correlated expression of enzymes involved in different steps of glycosylation such as sialyltransferases (*ST3GAL2*, *ST6GALNAC1*), beta-1,3-N-acetylglucosaminyltransferases (*B3GNT5*, *B3GNT3*), fucosyltransferases (*FUT3*, *FUT2*), and UDP-GalNAc transferase (*GALNT3*). *MUC4* was also associated with genes associated with cell signaling containing SH2 domain (Cbl proto-oncogene C (*CBLC*), signal transducing adaptor family member 2 (*STAP2*), dual adaptor of phosphotyrosine and 3-phosphoinositides 1 (*DAPP1*), SH2 domain containing 3A (*SH2D3A*), protein tyrosine kinase 6 (*PTK6*), growth factor receptor bound protein 7 (*GRB7*), fyn related Src family tyrosine kinase (*FRK*), tensin 4 (*TNS4*)) or SH3 domains (MET transcriptional regulator (*MACC1*), Rho GTPase activating protein 27 (*ARHGAP27*), tight junction protein 2 (*TJP2*), Rho guanine nucleotide exchange factor-5 and -16 (*ARHGEF5*, *ARHGEF16*), protein tyrosine kinase 6 (*PTK6*), EPS8 like 1 (*EPS8L1*), tight junction protein 3 (*TJP3*) and *FRK*). Finally, several genes encoding proteins with a SEA domain (*ADGRF1*, *ST14*, *MUC16*) were correlated with *MUC4* expression. Additionally, we analyzed protein–protein interactions of differentially expressed proteins with MUC4 with the String 10 tool. We showed that MUC4 is directly related with CEACAM5, CEACAM6, MUC16, MUC20 and glycosylation enzymes (ST3GAL2, B3GNT3, B3GNT5 and GALNT3) (Additional file 4: Figure S3). Altogether, we have identified genes with expression correlated with *MUC4* involved notably in cell adhesion, cell–cell junctions, glycosylation and cell signaling. In order to understand the association between the observed aberrant expression of *MUC4* and other molecular events, we explored the correlation between *MUC4* expression in CCLE and DNA methylation (RRBS) of the top genes correlated with MUC4. We observed that *MUC4* expression is negatively correlated with the methylation score of 16 out of 20 of the top genes (*LCN2, MUC20, STEAP4, WFDC2, GJB3, SH2D3A, RNF39, PRSS22, HS3ST1, GPR87, TACST2, FAM83A, LAMC2, B3GNT3, CLDN7*) (Fig. 4) suggesting that the association of *MUC4* and the correlated genes could be mediated by methylation regulation. Only *ADGRF1* RBBS is not correlated with *MUC4* mRNA level. *MUC16*, *SCEL* and *C1ORF116* scores were not available. Additionally we also evaluated the copy number variation association of the top genes with *MUC4* expression. We only observed a weak amplification of *MUC20* copy number (Pearson’s correlation = 0.13) and a weak deletion of *MUC16* copy number (Pearson’s correlation = − 0.14) suggesting that the relationship between MUC4 expression and copy number variation of top genes is unlikely (Additional file 5: Figure S4).

**Table 3:** Gene ontology clustering on genes correlated with MUC4 expression

Enrichment score	Gene ontology terms and annotations	Count	p value
7.08	Cell–cell adherens junction	18	1.4E−08
	Cadherin binding involved in cell–cell adhesion	17	2.0E−08
	Cell–cell adhesion	14	2.2E−06
5.44	Tight junction	10	6.6E−08
	Bicellular tight junction	10	1.4E−06
	Tight junction	9	8.1E−06
	Bicellular tight junction assembly	5	2.4E−04
4.67	Pleckstrin homology-like domain	17	2.6E−06
	Pleckstrin homology domain	13	9.3E−06
	Domain: PH	11	8.0E−05
	PH	12	1.1E−04
3.35	SH2 domain	8	9.1E−05
	Domain: SH2	7	2.3E−04
	SH2 domain	7	3.9E−04
	SH2	6	4.8E−03
3.34	Glycoprotein	64	6.0E−05
	Glycosylation site: N-linked (GlcNAc…)	61	1.1E−04
	Disulfide bond	44	6.4E−04
	Signal peptide	48	9.7E−04
	Disulfide bond	48	9.8E−04
	Signal	54	2.2E−03
2.76	Topological domain: cytoplasmic	53	8.1E−05
	Membrane	91	1.6E−04
	Transmembrane region	66	8.5E−04
	Topological domain: extracellular	42	9.2E−04
	Transmembrane helix	66	7.2E−03
	Transmembrane	66	7.7E−03
	Integral component of membrane	59	8.4E−02
2.6	Domain: SH3	9	1.9E−04
	SH3 domain	9	6.5E−04
	Src homology-3 domain	8	4.4E−03
	SH3	6	6.9E−02
2.48	Signal peptide	48	9.7E−04
	Secreted	31	2.0E−03
	Extracellular region	25	1.9E−02
2.43	Establishment of protein localization to plasma membrane	6	4.9E−05
	Cell adhesion molecule binding	5	3.0E−03
	Actin cytoskeleton	4	3.5E−01
2.32	Extracellular matrix organization	10	1.2E−04
	Epidermolysis bullosa, junctional, non-Herlitz type	3	2.8E−04
	Epidermolysis bullosa	4	2.8E−04
	Hemidesmosome assembly	3	5.7E−03
	ECM-receptor interaction	4	2.9E−02
	Focal adhesion	5	7.2E−02
	PI3K-Akt signaling pathway	4	5.0E−01
2.19	Serine protease	8	2.5E−04
	Peptidase S1, trypsin family, active site	7	3.9E−04
	Domain: peptidase S1	7	4.7E−04
	Active site: charge relay system	9	5.3E−04
	Peptidase S1	7	9.1E−04
	Trypsin-like cysteine/serine peptidase domain	7	1.3E−03
	Tryp_SPc	7	1.6E−03
	Extrinsic component of plasma membrane	4	1.7E−03
	Peptidase S1A, chymotrypsin-type	6	4.1E−03
	Serine-type endopeptidase activity	8	1.2E−02
	Serine-type peptidase activity	4	2.3E−02
	Protease	8	2.0E−01
	Zymogen	4	2.9E−01
	Proteolysis	7	3.5E−01
	Hydrolase	13	8.1E−01
1.74	CP2 transcription factor	3	1.3E−03
	Region of interest: transcription activation	3	3.5E−03
	Chromatin DNA binding	3	1.1E−01
	Sequence-specific DNA binding	8	2.3E−01
1.69	*O*-glycan processing	6	2.7E−04
	Glycosphingolipid biosynthesis—lacto and neolacto series	4	9.8E−04
	Protein glycosylation	6	4.7E−03
	Glycosyltransferase	7	1.8E−02
	Topological domain: lumenal	10	2.1E−02
	Golgi cisterna membrane	4	3.6E−02
	Signal-anchor	9	4.8E−02
	Golgi apparatus	12	1.0E−01
	Golgi membrane	9	2.0E−01
	Metabolic pathways	9	7.5E−01
1.51	Rho guanyl-nucleotide exchange factor activity	5	6.4E−03
	Regulation of Rho protein signal transduction	5	7.6E−03
	Dbl homology (DH) domain	4	2.9E−02
	Domain: DH	3	1.3E−01
	RhoGEF	3	1.6E−01

Gene list was retrieved from 881 samples of Cancer Cell Line Encyclopedia (Baretina, Nature 2012). 187 genes that are positively (n = 178) or negatively (n = 9) correlated with MUC4 expression were selected. Functional Annotation and gene clustering were performed using David Functional Annotation Tool (https://david.ncifcrf.gov/)

### MUC4 and patient survival

**Fig. 5:**
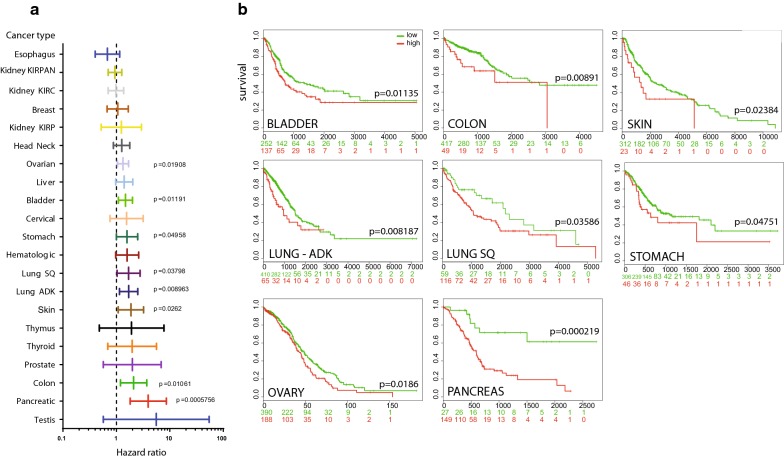
*MUC4* expression is associated with reduced overall survival of carcinoma. **a** Hazard ratio was calculated in population designated as MUC4 high risk and low risk (higher value of MUC4 for higher risk) by SurvExpress optimized algorithm in every cancer from TCGA datasets. **b** Overall survival values of MUC4 high and low risk groups in bladder cancer, colon cancer, lung adenocarcinoma, lung squamous carcinoma, ovarian cancer, skin cancer, stomach cancer, available in TCGA datasets. The numbers below horizontal axis represent the number of individuals not presenting the event of MUC4 high and low risk group along time

To establish a correlation between *MUC4* expression and patient survival, we have compared survival analysis and hazard ratio in population designated as MUC4 high risk and low risk in every organ from TCGA datasets (Table 4). We have used SurvExpress optimized algorithm that generates risk group by sorting prognostic index (higher value of MUC4 for higher risk). The algorithm splits the populations where the p-value testing the difference of *MUC4* expression is minimal [4]. Pancreatic cancer presented the most important hazard ratio for MUC4 (HR = 3.94 [CI 1.81–8.61] p = 0.0005756) (Fig. 5a). MUC4 high risk was also significantly associated with survival in bladder cancer (HR = 1.48), colon cancer (HR = 2.1), lung adenocarcinoma (HR = 1.7), lung squamous carcinoma (HR = 1.69), ovarian cancer (HR = 1.33), skin cancer (HR = 1.87) and stomach cancer (HR = 1.58) (Fig. 5a). Acute myeloid leukemia (HR = 1.59) and liver cancer (HR = 1.4) almost reach statistical significance. Other datasets did not show any statistically significant differences.

**Table 4:** Hazard-ratio and survival analysis of high and low risk in TCGA tumor databases

Database	N; low vs risk group	Hazard ratio [95% CI]	p value
Bladder–BLCA–TCGA–Bladder Urothelial Carcinoma–July 2016	N = 388; 251 vs 137	1.48 [1.09; 2]	*p = 0.01191*
Breast–BRCA–TCGA Breast invasive carcinoma–July 2016	N = 962; 831 vs 131	1.06 [0.67; 1.67]	p = 0.8038
Cervical–CESC–TCGA Cervical squamous cell carcinoma and endocervical adenocarcinoma July 2016	N = 191; 147 vs 44	1.55 [0.76; 3.17]	p = 0.2275
Colon–COADREAD–TCGA Colon and Rectum adenocarcinoma June 2016	N = 466; 417 vs 49	2.1 [1.19; 3.71]	*p = 0.01061*
Esophagus–ESCA–TCGA Esophageal carcinoma June 2016	N = 184; 137 vs 47	0.68 [0.4; 1.15]	p = 0.1468
Head–Neck–HNSC–TCGA Head and Neck squamous cell carcinoma June 2016	N = 502; 107 vs 395	1.26 [0.88; 1.78]	p = 0.204
Hematologic–Acute Myeloid Leukemia TCGA	N = 168; 146 vs 22	1.59 [0.97; 2.62]	p = 0.06818
Kidney–KIPAN–TCGA Kidney PAN cancer TCGA June 2016	N = 792; 555 vs 237	0.94 [0.7; 1.26]	p = 0.6711
Kidney–KIRC–TCGA–Kidney renal clear cell carcinoma	N = 415; 256 vs 159	0.98 [0.7; 1.37]	p = 0.9115
Kidney–KIRP–TCGA Kidney renal papillary cell carcinoma June 2016	N = 278; 248 vs 30	1.24 [0.52; 2.94]	p = 0.6322
Liver–TCGA–Liver–Cancer	N = 304; 137 vs 167	1.4 [0.97; 2.03]	p = 0.07012
Lung ADK–LUAD–TCGA–Lung adenocarcinoma June 2016	N = 475; 410 vs 65	1.7 [1.14; 2.52]	*p = 0.008963*
Lung Squamous–LUSC–TCGA–Lung squamous cell carcinoma June 2016	N = 175; 59 vs 116	1.69 [1.03; 2.78]	*p = 0.03798*
Ovarian–Ovarian serous cystadenocarcinoma TCGA	N = 578; 390 vs 188	1.33 [1.05; 1.69]	*p = 0.01908*
Pancreatic–PAAD–TCGA–Pancreatic adenocarcinoma	N = 176; 27 vs 149	3.94 [1.81; 8.61]	*p = 0.0005756*
Prostate–PRAD–TCGA–Prostate adenocarcinoma June 2016	N = 497; 328 vs 169	1.99 [0.57; 6.88]	p = 0.2793
Skin–SKCM–TCGA Skin Cutaneous Melanoma July 2016	N = 334; 312 vs 23	1.87 [1.08; 3.23]	*p = 0.0262*
Stomach–STAD–TCGA–Stomach adenocarcinoma June 2016	N = 352; 306 vs 46	1.58 [1; 2.51]	*p = 0.04958*
Testis–TGCT–TCGA–Testicular Germ Cell Tumors	N = 133; 93 vs 40	5.56 [0.57; 54.52]	p = 0.1407
Thymus–THYM–TCGA–Thymoma June 2016	N = 118; 90 vs 28	1.92 [0.48; 7.77]	p = 0.3588
Thyroid–THCA–TCGA–Thyroid carcinoma–June 2016	N = 247; 45 vs 202	1.98 [0.69; 5.64]	p = 0.2019

Hazard ratio and p-value were determined using SurvExpress tool (http://bioinformatica.mty.itesm.mx/SurvExpress). Risk groups were determined using the optimization algorithm (maximize) from the ordered prognostic index (higher values of MUC4 expression for higher risk). Statistical significant p-values are italicized

A significant reduction in patient’s survival was observed in bladder cancer (p = 0.01135), colon cancer (p = 0.00891), lung adenocarcinoma (p = 0.008187), lung squamous carcinoma (p = 0.03586), ovarian cancer (p = 0.0186), pancreatic cancer (p = 0.000219), skin cancer (p = 0.02384) and stomach cancer (p = 0.04751) as illustrated in Kaplan–Meier curves (Fig. 5b). Strikingly, pancreatic median survival was 593 days in *MUC4*^high^ cohort (n = 149) whereas the 50% survival was not reached in *MUC4*^low^ cohort (n = 27). In lung squamous carcinoma, the median survival of *MUC4*^high^ cohort (n = 116) was 1067 days whereas *MUC4*^low^ cohort (n = 59) presented a 2170 days median survival. It is interesting to note that the algorithm splits the population in two parts that were characterized as the most different regarding *MUC4* expression. Therefore, there are a modest number of *MUC4*^low^ PDAC or lung adenocarcinoma patients and a low number of *MUC4*^high^ colon or stomach cancer patients. A similar survival analysis was performed on pancreatic cancer by dividing the patient population in two equal parts (88 vs 88), *MUC4*^high^ harbored a decreased survival that was close to statistical significance (p = 0.06784) (not shown). Therefore, *MUC4* expression is associated with a poorer overall survival in different cancers including pancreatic cancer.

**Fig. 6:**
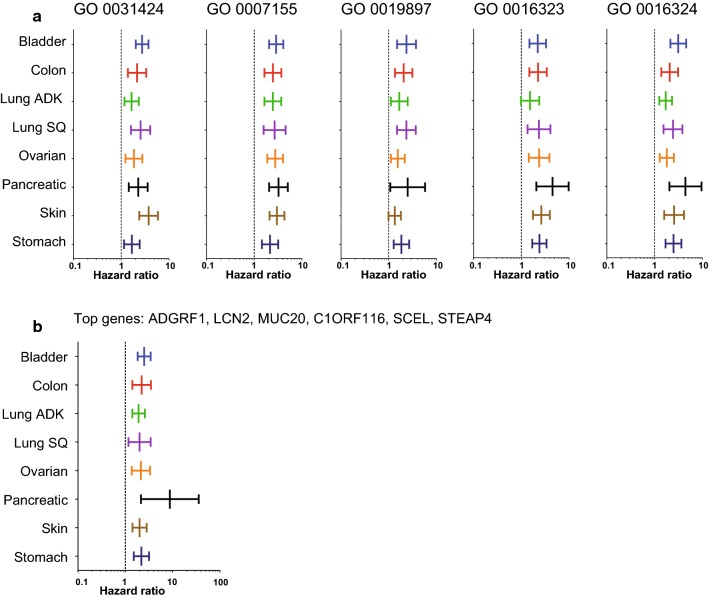
Hazard ratio of signatures defined by gene ontology terms and top-genes correlated with *MUC4*. **a** Hazard ratio was calculated in bladder cancer, colon cancer, lung adenocarcinoma, lung squamous carcinoma, ovarian cancer, pancreatic cancer, skin cancer and stomach cancer. The populations were defined according to GO term extracted from list of gene correlated with *MUC4* (GO 0031424: keratinization, GO 0007155: cell adhesion, GO 0019897: extrinsic component of plasma membrane, GO 0016323: basolateral plasma membrane and GO 0016324: apical plasma membrane). **b** A Hazard ratio was calculated in populations designated as high risk and low risk for top genes (*ADGRF1*, *LCN2*, *MUC20*, *C1ORF116*, *SCEL*, *STEAP4*) that harbored Pearson’s correlation with *MUC4* superior to 0.5

We also compared the survival and hazard ratio, in the same cancers whose survival is associated with MUC4 (bladder cancer, colon cancer, lung adenocarcinoma, lung squamous carcinoma, ovarian cancer, pancreatic cancer, skin cancer and stomach cancer), according to gene signatures corresponding to the five first gene ontology term from Additional file 3: Table S1 (GO 0031424: keratinization, GO 0007155: cell adhesion, GO 0019897: extrinsic component of plasma membrane, GO 0016323: basolateral plasma membrane and GO 0016324: apical plasma membrane) (Fig. 6a, Additional file 6: Table S2). These gene signatures were all significantly associated with survival in the TCGA dataset tested. The “keratinization” (GO 0031424) and “cell adhesion” (GO 0007155) signature are associated with HR comprised between 1.65 and 3.76 and between 2.15 and 3.23, respectively. The GO 0019897 signature is associated with weaker HR (1.55–2.30). “basolateral” (GO 0016323) and “apical plasma membrane” (GO 0016324) signatures harbor more increased HR (2.21–4.5 and 1.77–4.42, respectively) in these datasets.

We performed a similar analysis according to the top genes (*ADGRF1, LCN2, MUC20, C1ORF116, SCEL, STEAP4*) that harbored Pearson’s correlation with *MUC4* superior to 0.5 (Fig. 6b, Additional file 7: Table S3). This signature is associated with survival in all TCGA dataset tested (HR comprised between 1.91 and 8.77). Notably, pancreatic cancer harbored the strongest association with survival according to this signature (HR = 8.77 [CI 2.15–35.83]). Overall, these bigger signatures harbored higher hazard ratio compared to MUC4 alone.

### MUC4, MUC16 and MUC20 signature in cancer

**Fig. 7:**
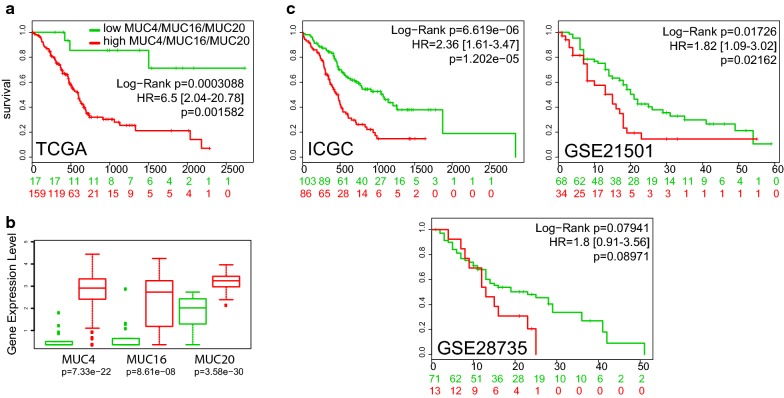
*MUC4*/*MUC16*/*MUC20* expression is associated with reduced overall survival of pancreatic adenocarcinoma. **a** Overall survival of *MUC4*/*MUC16*/*MUC20* high and low risk group in pancreatic cancer available in TCGA datasets. High risk and low risk cohorts were determined by SurvExpress optimized algorithm. Log rang test and Hazard ratio were calculated to compare both cohorts. **b** Box plot of *MUC4*, *MUC16* and *MUC20* expression and the corresponding p value testing the differences between high risk and low risk groups. **c** Overall survival of *MUC4*/*MUC16*/*MUC20* high and low risk groups in ICGC, Stratford (GSE21521) and Zhang (GSE 28735) datasets available in SurvExpress

Mucins have been proposed as potential biomarkers for carcinoma. Notably, previous work suggested that combination of mucins expression may be useful for early detection and evaluation of malignancy of pancreatobiliary neoplasms [24]. Moreover, MUC16/CA125 antigen is an already routinely used serum marker for the diagnosis of ovarian cancer [16]. Therefore, we decided to intentionally focus on the two other membrane bound mucins *MUC16* and *MUC20* that were correlated with expression of *MUC4*. We analyzed the survival curves of the high risk group (*MUC4*/*MUC16*/*MUC20*^high^, n = 159) and low risk group (*MUC4*/*MUC16*/*MUC20*^low^, n = 17) from the pancreas TCGA dataset. The *MUC4*/*MUC16*/*MUC20*^high^ risk group was associated with an increased hazard ratio (HR = 6.5 [2.04–20.78], p = 0.001582) and a shorter overall survival (p = 0.0003088) (Fig. 7a). Median survival was similar as in *MUC4*^high^ cohort (593 days). The *MUC4*/*MUC16*/*MUC20*^high^ group harbored a statistically significant increase of *MUC4*, *MUC16* and *MUC20* expression (Fig. 7b). We also analyzed overall survival in every other PDAC database available in Surexpress. We show that *MUC4*^high^ group was associated with a statistically significant reduced overall survival and increased hazard ratio in both ICGC and Stratford (GSE21501) cohorts (Fig. 7c). In Zhang cohort (GSE28735), *MUC4*^high^ group was associated with a reduced overall survival that was close to statistical significance (p = 0.08971). In other organs, the *MUC4*/*MUC16*/*MUC20*^high^ group was associated with an increased hazard ratio and reduced overall survival in bladder cancer, colon cancer, lung adenocarcinoma, lung squamous adenocarcinoma, skin cancer, stomach cancer (Additional file 8: Figure S5A). Notably, the *MUC4/MUC16/MUC20*^high^ group in colon cancer (HR = 2.26 [1.51–3.4]) showed a median survival of 1741 days whereas the low risk group did not reach the 50% survival. Similarly, the *MUC4/MUC16/MUC20*^high^ group in stomach cancer showed a median survival of 762 days whereas the low risk had a median survival of 1811 days. No significant difference was observed for ovarian cancer (p = 0.2081). Moreover, a reduced overall survival was observed in liver cancer (p = 0.04789) and acute myeloid leukemia (AML) (p = 0.02577) (Additional file 8: Figure S5B) in which we did not show any statistical difference when sorting the patients for MUC4 alone. Overall, we observed that *MUC4/MUC16/MUC20* signature harbored an increased hazard ratio compared with *MUC4* alone for pancreatic cancer and to a lower extent in bladder cancer, colon cancer, lung squamous cancer and stomach cancer.

**Fig. 8:**
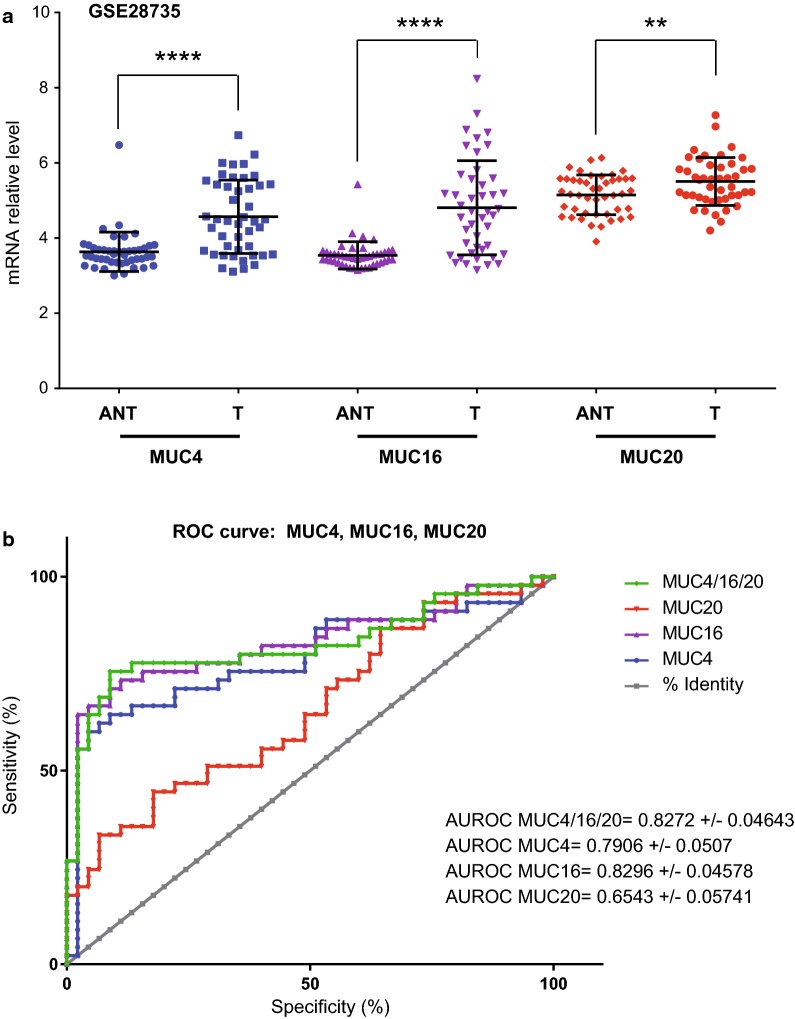
Expression and ROC curves of the *MUC4*/*MUC16*/*MUC20* signature in a pancreatic adenocarcinoma dataset. **a** *MUC4*, *MUC16* and *MUC20* mRNA expression was evaluated in GSE28735 dataset to analyze whether the mRNA level differed between normal and tumor tissues. Statistical analyses were performed using paired t-test (**** p < 0.0001, ** p < 0.01). **b** ROC curves and Area under ROC measurement (AUROC) of MUC4, MUC16, MUC20 and the combination in GSE28735 dataset

We analyzed *MUC4*, *MUC16 *and *MUC20* expression in pancreatic tumor (T) and paired adjacent non tumoral tissues (ANT) from GSE28735 (Fig. 6) and GSE16515 (not shown) datasets [25, 26]. We confirmed *MUC4* overexpression in tumor tissues (p < 0.0001). *MUC16* and *MUC20* mRNA level were also increased (p < 0.0001 and p = 0.0062) in tumor samples (Fig. 8a). As previously observed in CCLE dataset, *MUC4* expression was correlated with *MUC16* (p = 0.0006) and *MUC20* (p = 0.0621) in GSE28735 (Additional file 9: Figure S6). We also analyzed *MUC4*, *MUC16* and *MUC20* expression in datasets of other cancers (Additional file 10: Figure S7). *MUC4* expression is increased in bladder cancer vs ANT (GSE13507, p < 0.01). *MUC20* is increased in lung adenocarcinoma vs normal samples (GSE30219, p < 0.05). *MUC4 *and *MUC20* expression is increased in colorectal cancer vs normal mucosae (GSE40967, p < 0.01). *MUC16* and *MUC20* relative expression is increased in ovarian adenocarcinoma (GSE14407, p < 0.01 and p < 0.05 respectively). ROC curves of MUC4, MUC16, MUC20 and MUC4 + MUC16 + MUC20 combination were established using GSE28735 dataset. The combination of MUC4 + MUC16 + MUC20 produced a high specificity of 97.78% (88.23–99.94) and a mild sensitivity of 55.56% (40–70.36) (likelihood ratio = 25) (Fig. 8b). Similar results were obtained for GSE16515 with 93.75% specificity and 69.44% sensitivity (LR ± 11.11) (not shown). MUC16 AUROC was similar to that of MUC4 + MUC16 + MUC20 in GSE28735 dataset but harbored a lower specificity/sensitivity in GSE16515.

Altogether, this suggests that *MUC4/MUC16/MUC20*^high^ signature would be useful in stratification of patients with worst prognosis in several carcinoma and notably pancreatic, stomach and colon cancers.

## Discussion

The TCGA and the CCLE have provided a tremendous amount of publicly available data combining gene expression information related to clinical outcome. Web-based tools allow the scientific community to perform powerful large scale genomic analysis and propose new biomarkers or new therapeutic targets. In the present report, we analyzed *MUC4* expression systematically in all organs and confirmed its aberrant expression in associated carcinoma. We identified 187 genes for which the expression is correlated with *MUC4* expression. These genes are involved in cell adhesion, cell–cell junctions, glycosylation and cell signaling. *MUC4* was also correlated with *MUC16* and *MUC20* membrane-bound mucins. This combination is associated with a poorer overall survival in different cancers including pancreatic, colon and stomach cancers suggesting *MUC4*/*MUC16*/*MUC20* as a poor prognostic signature for these cancers.

Previous works have showed that MUC4 is altered in normal, premalignant and malignant epithelia of the digestive tract [27]. The mechanisms underlying this alteration of expression are diverse and involve regulators such as growth factors, cytokines, demethylation of promoters and miRNA [28–32]. In the present manuscript we also observe that MUC4 gene is amplified in 13% of cancer cell lines. We also found a mild correlation between alteration of *MUC4* copy number and *MUC4* expression suggesting that gene amplification could also mediate this *MUC4* aberrant expression. This kind of regulation is scarcely described in the literature. In TCGA, We confirmed that *MUC4* expression was observed mainly in human carcinomas including bladder, cervix, head and neck, lung, ovarian, pancreatic, prostate, stomach carcinomas. For most of these organs, *MUC4* high expression was associated with a poorer overall survival. *MUC4* is one of the most differentially expressed genes in pancreatic cancer that are thought to be potential clinical targets [33]. Recently, a meta-analysis based on 1900 patients from 18 studies showed that MUC4 overexpression was associated with tumor stage, tumor invasion and lymph node metastasis [34]. A worse overall survival was observed in MUC4-overexpressing patients with biliary tract carcinoma (HR 2.41), pancreatic cancer (HR 2.01), and colorectal cancer (HR 1.73). Using the TCGA cohorts, we extended this finding on lung adenocarcinoma, lung squamous carcinoma, ovarian cancer, skin cancer and stomach cancer. The authors noted that a limit of this meta-analysis was insufficient statistical power of some eligible studies. The large scale genomic approach of TCGA helps us to overcome this limitation. Based on available TCGA datasets, mucin mutation map was generated by cBioPortal Mutation Mapper [35]. *MUC4* mutations were notably observed in Kidney Clear Cell Renal Carcinoma (20–45%) and were correlated with survival outcomes. Rare mutations were described in the main overexpressing model that is pancreatic cancer. Because of the very large size of *MUC4* gene, probability of acquiring mutation could be increased. *MUC4* belongs to the most mutated genes upon stress exposure such as nicotine treatment or aging [36, 37]. The enrichment of mutation of *MUC4* could be related with the fact that the first risk factor of kidney cancer is smoking [38] and that kidney cancer diagnosis is occurring at elder ages (65 years) [39]. Pancreatic cancer shares these characteristics but harbors a very rare mutation occurrence (3%) suggesting that aging could be specific of cancers such as kidney or lung and that overexpression is more important for other cancers. So far, functional consequences of MUC4 mutation remain to be elucidated.

We and others have investigated MUC4 biological roles in various cancers such as pancreatic, ovarian, esophagus and lung cancers. MUC4 was shown to promote aggressiveness of tumors as it induces proliferation, migration, invasion, EMT, cell stemness and chemoresistance [9, 11–14]. In the present work, we showed that *MUC4* expression was correlated with genes, such as integrins cadherin-type proteins, involved in cell adhesion and cell–cell junctions. As a membrane-bound mucin, MUC4 is thought to act on cell–cell and cell-MEC interaction. Because of its huge extracellular domain that profoundly modifies steric hindrance, MUC4 may alter migration, invasion and adherence properties [40]. Rat homologue of MUC4, sialomucin complex (SMC), overexpression leads to suppression of cell adhesion [41]. Notably, MUC4 overexpression disrupts the adherens junctions and leads to partial delocalization of E-cadherin to the apical surface of the cell causing loss of cell polarity [42]. Moreover, interactions between MUC4 glycans and galectin-3 were shown to also mediate docking of circulating tumor cells to the surface of endothelial cells [43]. The alteration of cell adhesion induced by MUC4 is one of the first steps toward the metastatic process. MUC4 expression was also correlated with several genes encoding glycosylation enzymes or glycoproteins. This essential set of genes is involved in a wide set of cellular function including cell adhesion, barrier role, interaction with selection of endothelial cells or regulation of cell signaling [5, 44]. The glycan-associated antigens are commonly associated with patient survival of gastrointestinal cancer [45]. Alteration of MUC4 glycosylation is proposed to play a substantial role in binding properties mediated by the extracellular subunit of MUC4 and the NIDO domain [46]. One should note that the expression of these genes is correlated with MUC4. However, a direct regulatory mechanism remains to be demonstrated in future studies.

In order to regulate these major biological properties, MUC4 has been commonly associated with cell signaling alteration and notably MAPK, NF-kB, or FAK signaling pathways. Interestingly, we observed that MUC4 expression is highly correlated with proteins containing Src Homology 2 (SH2) domain or Src Homology 3 (SH3) domains. Intracellular adaptor signaling proteins family is characterized by one SH2 and at least one SH3 domain and is crucial for effective integrating of intracellular and extracellular stimuli [47].

It is interesting to note that *MUC4* expression is not correlated with MUC1 that is a major membrane-bound mucin commonly overexpressed in cancer [48, 49]. In the US, it was estimated that 900 000 cancers, out of 1 400 000, harbor overexpression of MUC1 highlighting its attractiveness as a therapeutic target. This could be explained by different regulatory mechanisms such as different signaling pathways or different miRNA regulating the two mucins.

MUC16 is the peptide part to the CA125 serum marker for ovarian cancer [50]. MUC16 is a very large mucin (22 000 amino acid (aa)) that is heavily glycosylated and facilitates ovarian cancer. MUC20 is a small mucin (500 aa) mostly expressed in renal proximal tube and that is deregulated in several cancers such as colorectal or ovarian cancers where it favors aggressiveness [17, 18]. MUC16/CA125 is routinely used in clinics unlike MUC4 and MUC20. In the present manuscript, we showed that expression of *MUC16* and *MUC20* are positively correlated with *MUC4* and that the *MUC4*/*MUC16*/*MUC20*^high^ combinatory expression is associated with an increased hazard ratio and reduced overall survival suggesting a potential for this signature as a prognostic marker for several carcinomas and notably pancreatic, stomach and colon cancer. Biomarkers for pancreatic cancer are needed for detection and evaluation of response to therapy [51]. Unfortunately, the marker currently used (CA19.9) lacks sensitivity or specificity to be used in cancer diagnosis. Similarly established biomarkers with adequate sensitivity and specificity are lacking for gastric cancer [52]. The need of biomarkers is less urgent for colorectal cancer since several predictive/prognostic/diagnostic biomarkers have been described [53].

The present work highlights the relationship between MUC4/MUC16/MUC20 expression and overall survival. This signature could be proposed as a prognostic marker. Moreover, MUC4 is expressed in the earliest stage (PanIN1A) of pancreatic cancer but is not specific enough. The potential of the combination *MUC4*/*MUC16*/*MUC20* as a diagnosis marker is not known and remains to be investigated in the future. Moreover, development of unsupervised algorithm will allow the identification of new non intentional bigger signatures leading to better prognostic and predictive performances. Genome wide computational unsupervised procedures from discovery datasets will help to determine hypothesis signature. The signature will be subsequently validated on a number of independents datasets. Thus, multi-platform analysis using TCGA datasets helped to characterize the complex molecular landscape of PDAC [54]. Another meta-analysis approach based on PDAC datasets allowed the identification of a 5 genes classifier signature (*TMPRSS4*, *AHNAK2*, *POSTN*, *ECT2*, *SERPINB5*) with 95% sensitivity and 89% specificity in discriminating PDAC from non-tumor samples [55]. Interestingly, *TMPRSS4* and *SERPINB5* are two genes belonging to the gene list correlated with *MUC4* expression.

## Conclusion

We analyzed *MUC4* expression systematically in all organs in TCGA and CCLE large scale databases and confirmed its aberrant expression in associated carcinoma and the MUC4 impact on patient’s survival. Moreover, 187 genes (involved in cell adhesion, cell–cell junctions, glycosylation and cell signaling) were correlated with MUC4. Among them, *MUC16* and *MUC20* membrane-bound mucins and their combination *MUC4/MUC16/MUC20* is associated with a poorer overall survival in different cancers including pancreatic, colon and stomach cancers suggesting *MUC4*/*MUC16*/*MUC20* as a poor prognostic signature for these cancers. This potential as new biomarkers remains to be investigated in the future.

## Additional files

**Additional file 1: Figure S1.** MUC4 Oncoprint in Cancer Cell Line Encyclopedia. MUC4 alterations were explored in Cancer Cell Line Encyclopedia dataset using cBioPortal webtool. The oncoprint represents the amplification, deletion, up regulation or in frame mutation.

**Additional file 2: Figure S2.** *MUC4* expression in normal tissues. *MUC4* expression was analyzed with https://gtexportal.org. Expression is shown as log10 of RKPM (read per kilobases of transcript per million map reads). Boxplot are shown as median and 25/75% percentile. Outliers are represented as points.

**Additional file 3: Table S1.** Ontology of genes correlated with *MUC4* expression. Gene list was retrieved from 881 samples of Cancer Cell Line Encyclopedia (Novartis/Broad, Nature 2012). 187 genes that are positively (n = 178) or negatively (n = 9) correlated with MUC4 expression were selected. Functional Annotation was performed using David Functional Annotation Tool.

**Additional file 4: Figure S3.** Interaction network of the proteins correlated with *MUC4* expression. Interacting proteins were determined by String 10 tool and are represented by nodes. Edges represent a relationship between two nodes (known interaction from curated databases or experimentally determined; predicted interaction from gene neighborhood, gene fusion or co-occurrence; textmining; co-expression; protein homology). The obtained network was divided in 3 clusters by k-means clustering.

**Additional file 5: Figure S4.** Correlation of *MUC4* expression and copy numbers of genes correlated with *MUC4*. The top genes were defined as genes harboring Pearson’s correlation higher than 0.5 with *MUC4* expression. *MUC4* mRNA expression and log2 copy number of *ADGRF1*, *LCN2, MUC20, C1ORF116, STEAP4, SCEL, MUC16* were extracted using (https://portals.broadinstitute.org/ccle).

**Additional file 6: Table S2.** Hazard-ratio and survival analysis of most significant genes clustered in GO term associated with *MUC4* expression in TCGA tumor databases. Hazard ratio and p-value were determined using SurvExpress tool (http://bioinformatica.mty.itesm.mx/SurvExpress). Risk groups were sorted depending on the major GO term GO 0031424, GO 00071555, GO 0019897, GO 0016323 and GO 0016324 using the optimization algorithm (maximize) from the ordered prognostic.

**Additional file 7: Table S3.** Hazard-ratio and survival analysis of top genes associated with *MUC4* expression in TCGA tumor databases. Hazard ratio and p-value were determined using SurvExpress tool (http://bioinformatica.mty.itesm.mx/SurvExpress). Risk groups were defined using the optimization algorithm (maximize) from the ordered prognostic. Selected genes (*ADGRF1*, *LCN2*, *MUC20*, *C1ORF116*, *SCEL*, *STEAP4*) harbored Pearson’s correlation with *MUC4* > 0.5.

**Additional file 8: Figure S5.** Overall survival of MUC4/MUC16/MUC20 high and low risk groups in cancer datasets available in TCGA. (A) Overall survival of *MUC4*/*MUC16*/*MUC20* high and low risk groups in bladder cancer, colon cancer, lung adenocarcinoma, lung squamous adenocarcinoma, skin cancer and stomach cancer. High risk and low risk cohorts were determined by SurvExpress optimized algorithm. Log rang test and Hazard ratio were calculated to compare both cohorts. The numbers below horizontal axis represent the number of individuals not presenting the event of *MUC4* high and low risk group along time. (B**)** Overall survival of *MUC4*/*MUC16*/*MUC20* high and low risk group in liver and acute myeloid leukemia (AML).

**Additional file 9: Figure S6.** *MUC4*-*MUC16* and *MUC4*-*MUC20* correlation of mRNA expression in 45 tumor tissues of GSE28735 PDAC dataset.

**Additional file 10: Figure S7.** *MUC4*, *MUC16* and *MUC20* expression in bladder, colorectal, lung, stomach, skin and ovarian cancer datasets. *MUC4*, *MUC16* and *MUC20* mRNA expression was evaluated in datasets to analyze whether the mRNA level differed between normal and tumor tissues. (A) GSE13507 contains 165 bladder cancer and 58 ANT samples. (B) GSE30219 contains 14 normal lung, 85 adenocarcinomas and 61 squamous cancer samples. (C) GSE40967 contains 566 colorectal cancers and 19 normal mucosae. (D) GSE27342 contains 80 tumors and 80 paired ANT tissues. (E) GSE4587 contains 2 normal, 2 melanomas and 2 metastatic melanomas. (F) GSE14407 contains 12 ovarian adenocarcinomas and 12 normal ovary samples. Statistical analyses were performed using paired t-test (*p<0.05, **p<0.01).

## Abbreviations

AUROC: area under receiving operator characteristic
CCLE: cancer cell line encyclopedia
HR: hazard ratio
PDAC: pancreatic ductal adenocarcinoma
ROC: receiving operator characteristic
TCGA: the cancer genome atlas
