# Supplementary figures and images for: Integrative analysis of the cancer genome atlas and cancer cell lines encyclopedia large-scale genomic databases: MUC4/MUC16/MUC20 signature is associated with poor survival in human carcinomas
Source: J Transl Med. 2018 Sep 20;16:259. doi: 10.1186/s12967-018-1632-2 (PMC6149062; doi:10.1186/s12967-018-1632-2)

Additional file 1: Figure S1

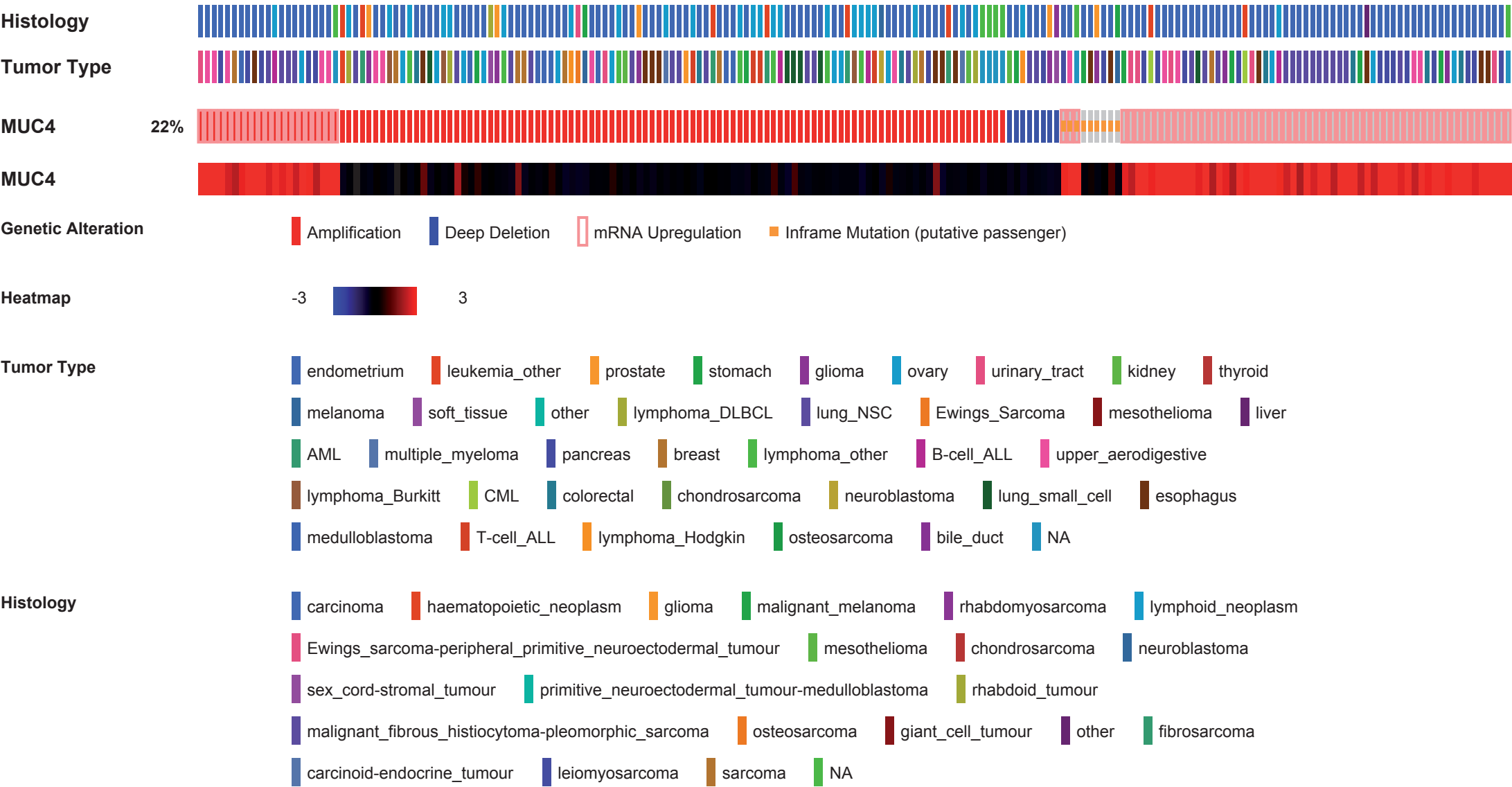

Supplement: Supplementary file 1 — Additional file 1: Figure S1. MUC4 Oncoprint in Cancer Cell Line Encyclopedia. MUC4 alterations were explored in Cancer Cell Line Encyclopedia dataset using cBioPortal webtool. The oncoprint represents the amplification, deletion, up regulation or in frame mutation. [file 12967_2018_1632_MOESM1_ESM.pdf]

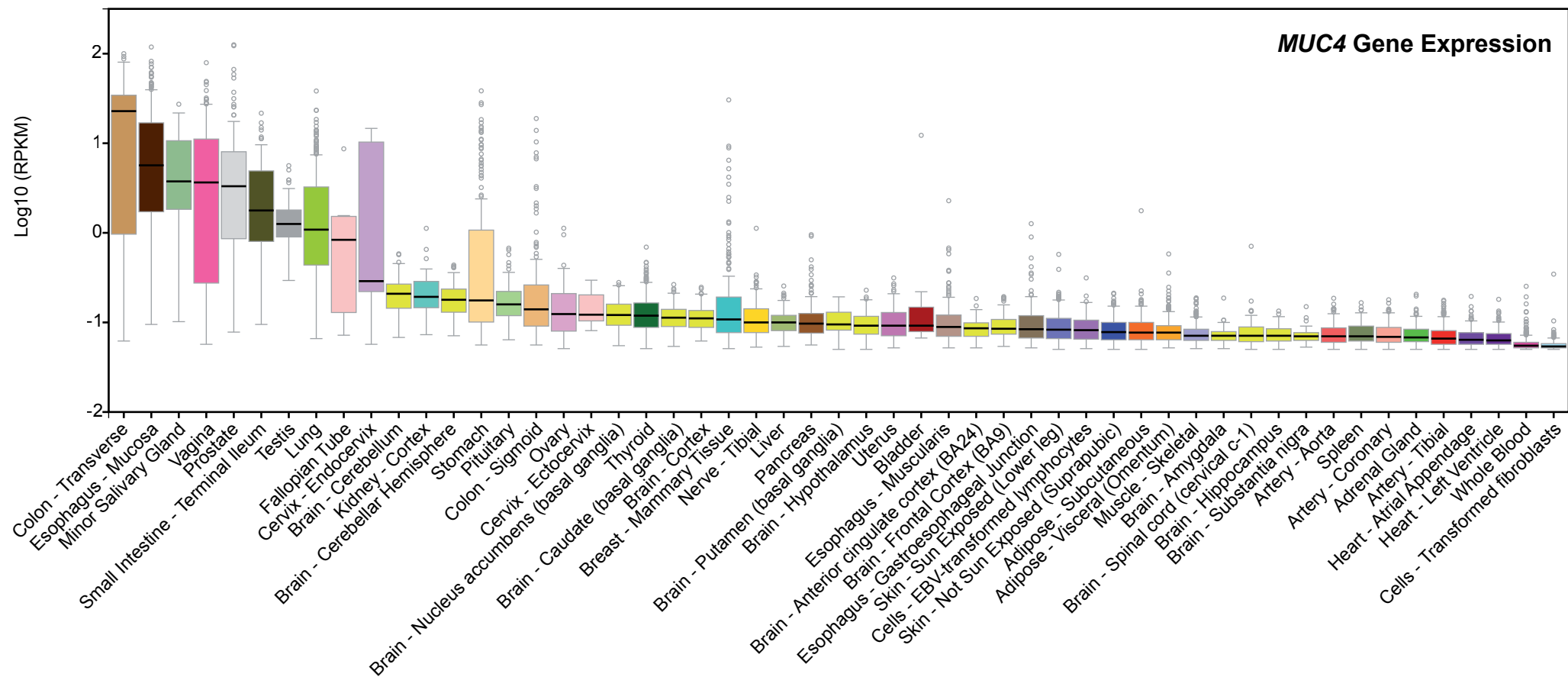

Supplement: Supplementary file 2 — Additional file 2: Figure S2. MUC4 expression in normal tissues. MUC4 expression was analyzed with https://gtexportal.org. Expression is shown as log10 of RKPM (read per kilobases of transcript per million map reads). Boxplot are shown as median and 25/75% percentile. Outliers are represented as points. [file 12967_2018_1632_MOESM2_ESM.pdf]

Additional file 4: Figure S3

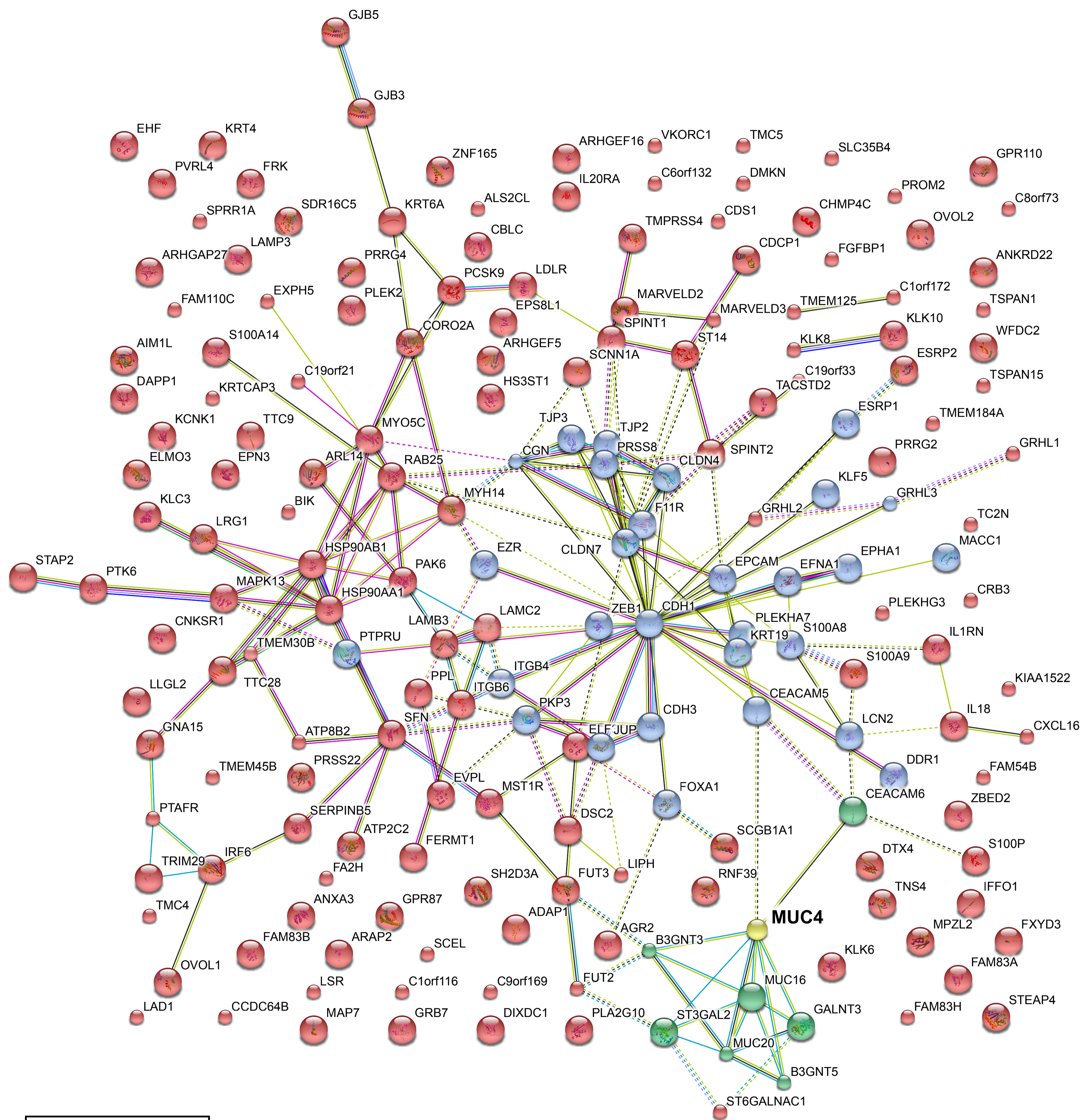

Supplement: Supplementary file 4 — Additional file 4: Figure S3. Interaction network of the proteins correlated with MUC4 expression. Interacting proteins were determined by String 10 tool and are represented by nodes. Edges represent a relationship between two nodes (known interaction from curated databases or experimentally determined; predicted interaction from gene neighborhood, gene fusion or co-occurrence; textmining; co-expression; protein homology). The obtained network was divided in 3 clusters by k-means clustering. [file 12967_2018_1632_MOESM4_ESM.pdf]

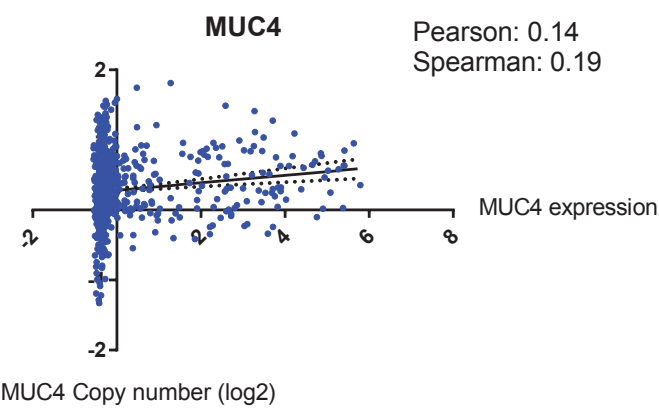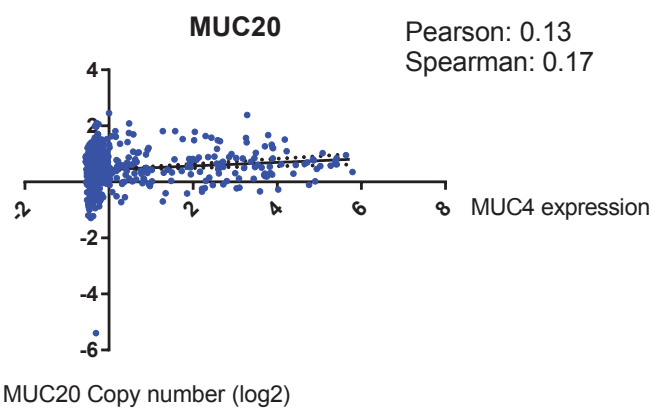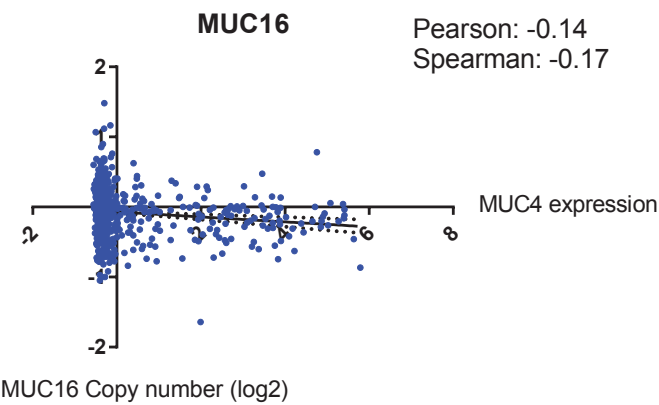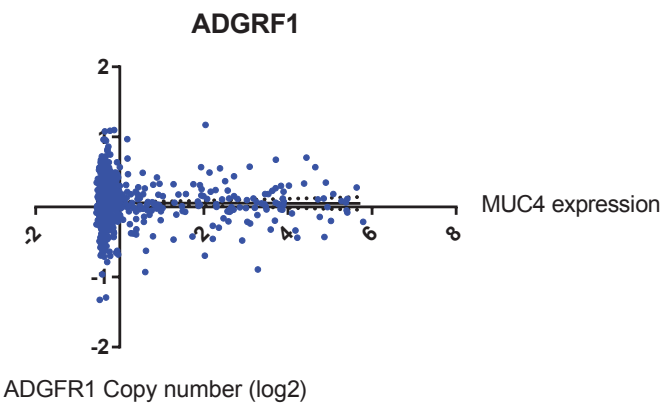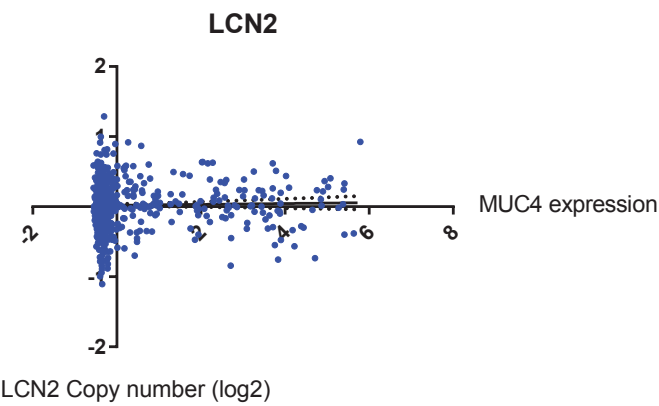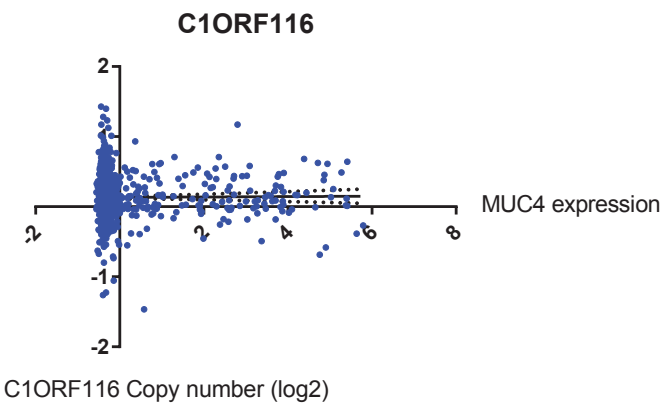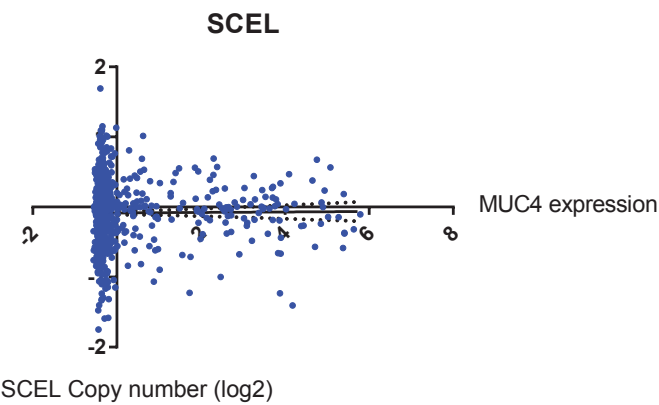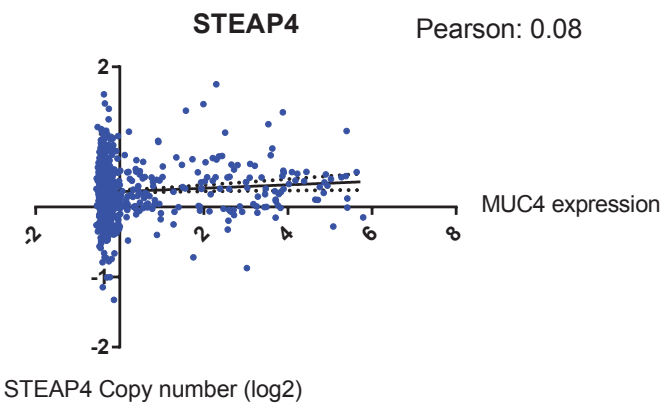

Supplement: Supplementary file 5 — Additional file 5: Figure S4. Correlation of MUC4 expression and copy numbers of genes correlated with MUC4. The top genes were defined as genes harboring Pearson’s correlation higher than 0.5 with MUC4 expression. MUC4 mRNA expression and log2 copy number of ADGRF1, LCN2, MUC20, C1ORF116, STEAP4, SCEL, MUC16 were extracted using (https://portals.broadinstitute.org/ccle). [file 12967_2018_1632_MOESM5_ESM.pdf]

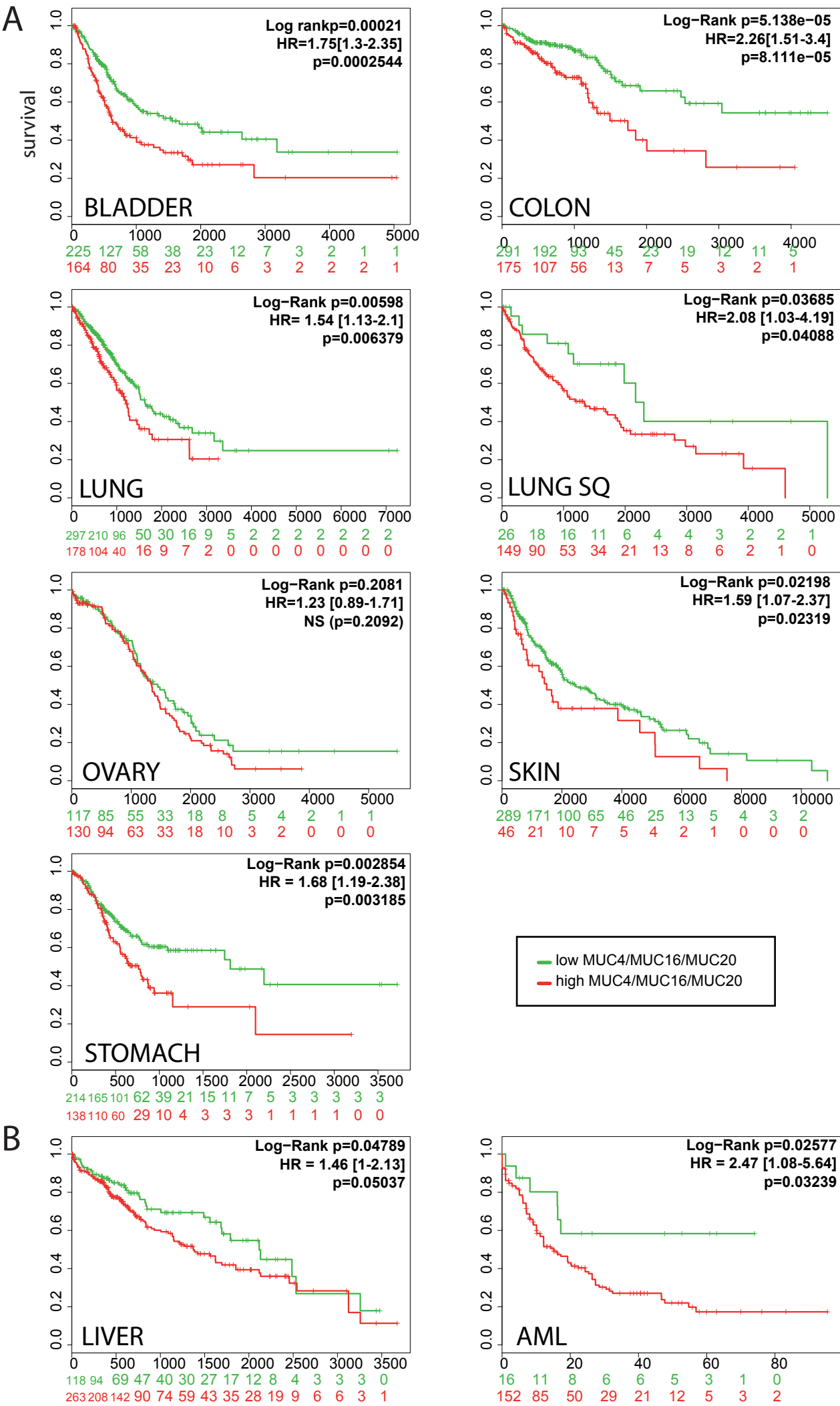

Supplement: Supplementary file 8 — Additional file 8: Figure S5. Overall survival of MUC4/MUC16/MUC20 high and low risk groups in cancer datasets available in TCGA. (A) Overall survival of MUC4/MUC16/MUC20 high and low risk groups in bladder cancer, colon cancer, lung adenocarcinoma, lung squamous adenocarcinoma, skin cancer and stomach cancer. High risk and low risk cohorts were determined by SurvExpress optimized algorithm. Log rang test and Hazard ratio were calculated to compare both cohorts. The numbers below horizontal axis represent the number of individuals not presenting the event of MUC4 high and low risk group along time. (B) Overall survival of MUC4/MUC16/MUC20 high and low risk group in liver and acute myeloid leukemia (AML). [file 12967_2018_1632_MOESM8_ESM.pdf]

# GSE28735

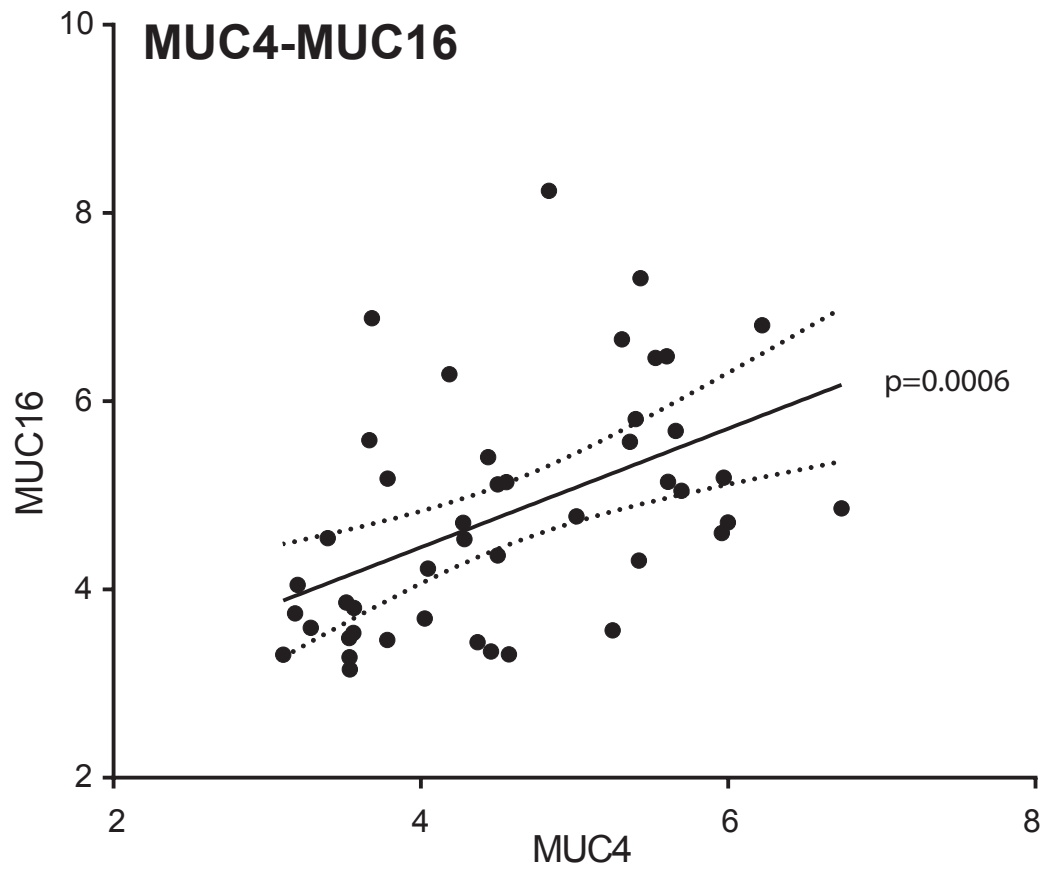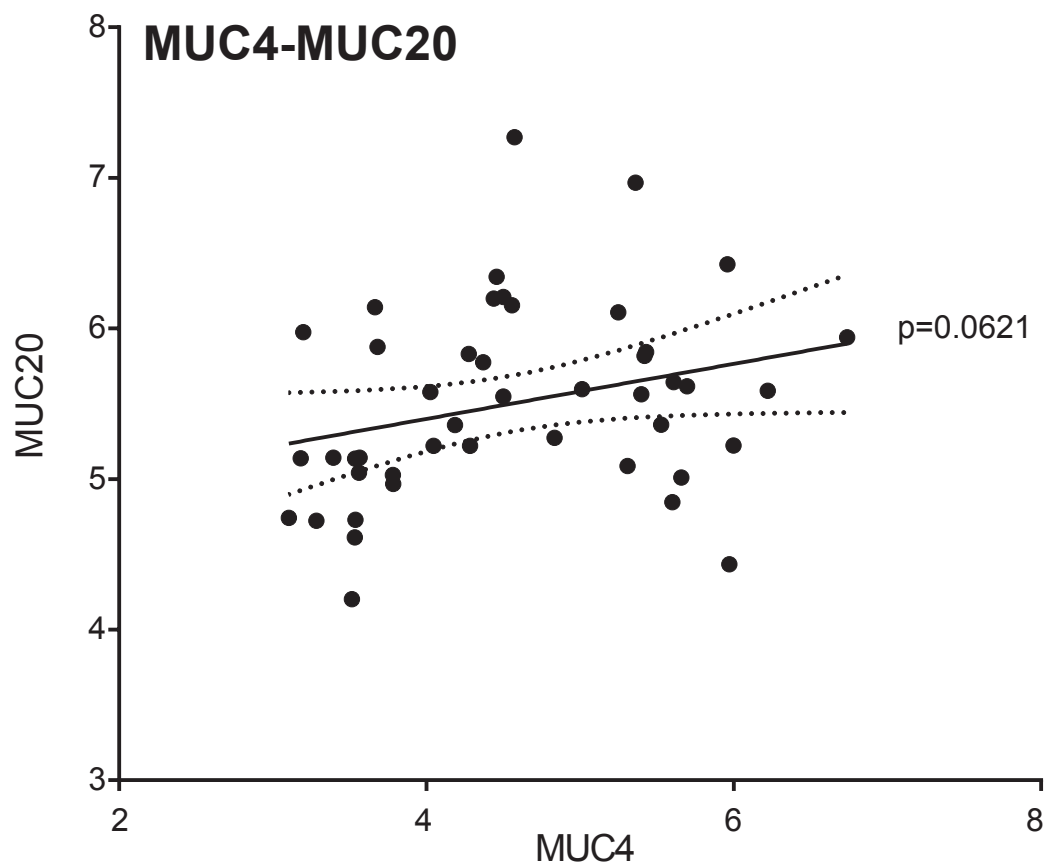

Supplement: Supplementary file 9 — Additional file 9: Figure S6. MUC4-MUC16 and MUC4-MUC20 correlation of mRNA expression in 45 tumor tissues of GSE28735 PDAC dataset. [file 12967_2018_1632_MOESM9_ESM.pdf]

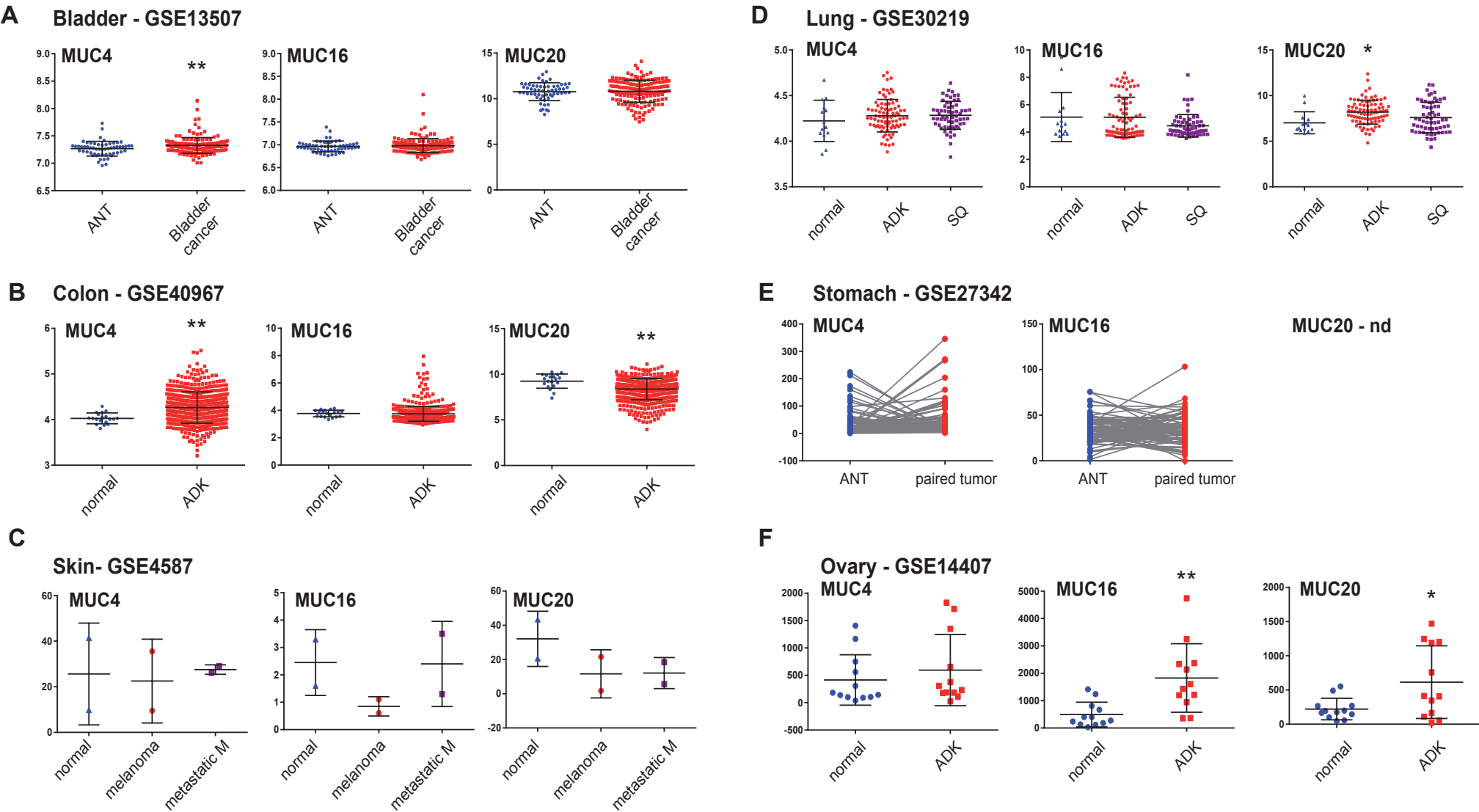

Supplement: Supplementary file 10 — Additional file 10: Figure S7. MUC4, MUC16 and MUC20 expression in bladder, colorectal, lung, stomach, skin and ovarian cancer datasets. MUC4, MUC16 and MUC20 mRNA expression was evaluated in datasets to analyze whether the mRNA level differed between normal and tumor tissues. (A) GSE13507 contains 165 bladder cancer and 58 ANT samples. (B) GSE30219 contains 14 normal lung, 85 adenocarcinomas and 61 squamous cancer samples. (C) GSE40967 contains 566 colorectal cancers and 19 normal mucosae. (D) GSE27342 contains 80 tumors and 80 paired ANT tissues. (E) GSE4587 contains 2 normal, 2 melanomas and 2 metastatic melanomas. (F) GSE14407 contains 12 ovarian adenocarcinomas and 12 normal ovary samples. Statistical analyses were performed using paired t-test (*p<0.05, **p<0.01). [file 12967_2018_1632_MOESM10_ESM.pdf]
