# Supplementary material for: Integrative analysis of the cancer genome atlas and cancer cell lines encyclopedia large-scale genomic databases: MUC4/MUC16/MUC20 signature is associated with poor survival in human carcinomas
Source: J Transl Med. 2018 Sep 20;16:259. doi: 10.1186/s12967-018-1632-2 (PMC6149062; doi:10.1186/s12967-018-1632-2)
Supplement: Supplementary file 3 — Additional file 3: Table S1. Ontology of genes correlated with MUC4 expression. Gene list was retrieved from 881 samples of Cancer Cell Line Encyclopedia (Novartis/Broad, Nature 2012). 187 genes that are positively (n = 178) or negatively (n = 9) correlated with MUC4 expression were selected. Functional Annotation was performed using David Functional Annotation Tool. [file 12967_2018_1632_MOESM3_ESM.docx]

**Additional file 3: Table S1: Ontology of genes correlated with MUC4 expression.** Gene list was retrieved from 881 samples of Cancer Cell Line Encyclopedia (Novartis/Broad, Nature 2012). 187 genes that are positively (n=178) or negatively (n=9) correlated with *MUC4* expression were selected. Functional Annotation was performed using David Functional Annotation Tool (<https://david.ncifcrf.gov/>).

| Category | Term | Count | % | PValue | Genes |
| --- | --- | --- | --- | --- | --- |
| UP_KEYWORDS | **Cell adhesion** | 13 | 7,0 | **0,0011** | MPZL2, FERMT1, ITGB4, CDH1, PTPRU, CDH3, EPHA1, JUP, LAMB3, PKP3, ITGB6, DSC2, LAMC2 |
| GOTERM_BP_DIRECT | **GO:0031424~keratinization** | 5 | 2,7 | **0,0011** | EVPL, SPRR1A, PPL, SFN, CDH3 |
| INTERPRO | **IPR007604:CP2 transcription factor** | 3 | 1,6 | **0,0013** | GRHL3, GRHL1, GRHL2 |
| INTERPRO | **IPR009003:Trypsin-like cysteine/serine peptidase domain** | 7 | 3,8 | **0,0013** | KLK6, PRSS8, KLK8, ST14, KLK10, PRSS22, TMPRSS4 |
| SMART | **SM00020:Tryp_SPc** | 7 | 3,8 | **0,0016** | KLK6, PRSS8, KLK8, ST14, KLK10, PRSS22, TMPRSS4 |
| GOTERM_BP_DIRECT | **GO:0007155~cell adhesion** | 13 | 7,0 | **0,0016** | JUP, DDR1, F11R, LAMB3, ITGB6, ITGB4, FERMT1, DSC2, LAMC2, PTPRU, CDH3, GRHL2, MUC16 |
| GOTERM_CC_DIRECT | **GO:0019897~extrinsic component of plasma membrane** | 4 | 2,2 | **0,0017** | PRSS8, EPN3, ST14, PRSS22 |
| GOTERM_CC_DIRECT | **GO:0016323~basolateral plasma membrane** | 8 | 4,3 | **0,0017** | EPCAM, CLDN7, PROM2, EZR, MARVELD2, ST14, MAP7, CEACAM5 |
| GOTERM_CC_DIRECT | **GO:0016324~apical plasma membrane** | 10 | 5,4 | **0,0020** | EPCAM, PROM2, EZR, CLDN4, MAL2, MARVELD2, MUC20, CRB3, KCNK1, SCNN1A |
| UP_KEYWORDS | **Secreted** | 31 | 16,8 | **0,0020** | ADGRF1, IL18, S100A9, CDCP1, SFN, SCGB1A1, PRSS8, LAMB3, ST3GAL2, LRG1, DMKN, PCSK9, KLK10, FGFBP1, KLK6, KLK8, LAD1, PLA2G10, IL1RN, MUC20, SPINT1, LCN2, DDR1, SERPINB5, CXCL16, LIPH, LAMC2, PRSS22, AGR2, WFDC2, MUC16 |
| UP_KEYWORDS | **Signal** | 54 | 29,2 | **0,0022** | CLDN7, MPZL2, ADGRF1, CDCP1, LSR, SCGB1A1, EPCAM, SPINT2, DMKN, VKORC1, CEACAM6, CEACAM5, KLK10, F11R, SPINT1, PTPRU, TNS4, DDR1, TACSTD2, IL20RA, LAMC2, MST1R, MTFR1L, WFDC2, EPS8L1, FXYD3, HS3ST1, ITGB4, CDH1, CDH3, PRSS8, LAMB3, PRRG4, LRG1, PRRG2, ITGB6, PCSK9, FUT2, FGFBP1, KLK6, KLK8, PLA2G10, IL1RN, MUC20, CRB3, EPHA1, LCN2, LAMP3, PROM2, CXCL16, LIPH, DSC2, AGR2, PRSS22 |
| GOTERM_CC_DIRECT | **GO:0005615~extracellular space** | 25 | 13,5 | **0,0023** | KLK6, KLK8, IL18, S100A9, IL1RN, SPINT1, SFN, SCGB1A1, LCN2, PRSS8, DDR1, EZR, SERPINB5, TACSTD2, LRG1, CXCL16, ST14, LIPH, CEACAM6, PCSK9, LAMC2, AGR2, FGFBP1, WFDC2, MUC16 |
| INTERPRO | **IPR012461:Protein of unknown function DUF1669** | 3 | 1,6 | **0,0023** | FAM83B, FAM83A, FAM83H |
| INTERPRO | **IPR014868:Cadherin prodomain** | 3 | 1,6 | **0,0023** | DSC2, CDH1, CDH3 |
| GOTERM_BP_DIRECT | **GO:0016337~single organismal cell-cell adhesion** | 6 | 3,2 | **0,0029** | JUP, MPZL2, PKP3, CDH1, PTPRU, CDH3 |
| GOTERM_MF_DIRECT | **GO:0050839~cell adhesion molecule binding** | 5 | 2,7 | **0,0030** | JUP, CLDN7, EZR, PKP3, CDH1 |
| SMART | **SM01055:SM01055** | 3 | 1,6 | **0,0030** | DSC2, CDH1, CDH3 |
| UP_SEQ_FEATURE | **region of interest:Transcription activation** | 3 | 1,6 | **0,0035** | GRHL3, GRHL1, GRHL2 |
| GOTERM_BP_DIRECT | **GO:0003334~keratinocyte development** | 3 | 1,6 | **0,0039** | KDF1, EXPH5, SFN |
| INTERPRO | **IPR001314:Peptidase S1A, chymotrypsin-type** | 6 | 3,2 | **0,0041** | KLK6, PRSS8, KLK8, KLK10, PRSS22, TMPRSS4 |
| GOTERM_BP_DIRECT | **GO:0043588~skin development** | 4 | 2,2 | **0,0042** | JUP, OVOL1, GJB3, ITGB4 |
| INTERPRO | **IPR001452:Src homology-3 domain** | 8 | 4,3 | **0,0044** | FRK, PTK6, ARHGEF5, ARHGEF16, ARHGAP27, TJP3, TJP2, EPS8L1 |
| GOTERM_BP_DIRECT | **GO:0006486~protein glycosylation** | 6 | 3,2 | **0,0047** | ST3GAL2, B3GNT5, FUT3, B3GNT3, FUT2, ST6GALNAC1 |
| GOTERM_CC_DIRECT | **GO:0030054~cell junction** | 12 | 6,5 | **0,0047** | F11R, KDF1, CGN, PLEKHA7, ARHGEF5, ITGB4, FERMT1, CRB3, CDH1, KCNK1, S100A14, TJP2 |
| SMART | **SM00252:SH2** | 6 | 3,2 | **0,0048** | TNS4, FRK, SH2D3A, DAPP1, PTK6, GRB7 |
| GOTERM_BP_DIRECT | **GO:0031581~hemidesmosome assembly** | 3 | 1,6 | **0,0057** | LAMB3, ITGB4, LAMC2 |
| GOTERM_BP_DIRECT | **GO:0030216~keratinocyte differentiation** | 5 | 2,7 | **0,0061** | EVPL, SPRR1A, IRF6, ST14, SCEL |
| GOTERM_BP_DIRECT | **GO:0001843~neural tube closure** | 5 | 2,7 | **0,0064** | SPINT2, ST14, SPINT1, GRHL3, GRHL2 |
| GOTERM_MF_DIRECT | **GO:0005089~Rho guanyl-nucleotide exchange factor activity** | 5 | 2,7 | **0,0064** | PLEKHG3, ARHGEF5, ARHGEF16, EPS8L1, ALS2CL |
| UP_KEYWORDS | **Transmembrane helix** | 66 | 35,7 | **0,0072** | CLDN7, STEAP4, MPZL2, ADGRF1, TSPAN1, CLDN4, CDCP1, GPR87, LSR, EPCAM, ST3GAL2, SPINT2, VKORC1, ATP8B2, F11R, FA2H, SPINT1, PTPRU, CDS1, KRTCAP3, ST6GALNAC1, DDR1, ATP2C2, TACSTD2, IL20RA, ST14, TMEM184A, MST1R, PTAFR, GALNT3, FXYD3, MAL2, ITGB4, CDH1, CDH3, PRSS8, PRRG4, B3GNT5, TMEM125, MARVELD2, PRRG2, MARVELD3, ITGB6, SLC35B4, FUT3, TMEM30B, SDR16C5, B3GNT3, FUT2, SCNN1A, TMEM45B, TMC5, TMC4, GJB3, CRB3, GJB5, TSPAN15, KCNK1, EPHA1, TMPRSS4, PROM2, LAMP3, CXCL16, DSC2, BIK, MUC16 |
| GOTERM_CC_DIRECT | **GO:0031410~cytoplasmic vesicle** | 8 | 4,3 | **0,0076** | F11R, ATP2C2, PROM2, MARVELD2, MARVELD3, ARL14, DSC2, RAB25 |
| GOTERM_BP_DIRECT | **GO:0035023~regulation of Rho protein signal transduction** | 5 | 2,7 | **0,0076** | PLEKHG3, ARHGEF5, ARHGEF16, EPS8L1, ALS2CL |
| UP_KEYWORDS | **Transmembrane** | 66 | 35,7 | **0,0077** | CLDN7, STEAP4, MPZL2, ADGRF1, TSPAN1, CLDN4, CDCP1, GPR87, LSR, EPCAM, ST3GAL2, SPINT2, VKORC1, ATP8B2, F11R, FA2H, SPINT1, PTPRU, CDS1, KRTCAP3, ST6GALNAC1, DDR1, ATP2C2, TACSTD2, IL20RA, ST14, TMEM184A, MST1R, PTAFR, GALNT3, FXYD3, MAL2, ITGB4, CDH1, CDH3, PRSS8, PRRG4, B3GNT5, TMEM125, MARVELD2, PRRG2, MARVELD3, ITGB6, SLC35B4, FUT3, TMEM30B, SDR16C5, B3GNT3, FUT2, SCNN1A, TMEM45B, TMC5, TMC4, GJB3, CRB3, GJB5, TSPAN15, KCNK1, EPHA1, TMPRSS4, PROM2, LAMP3, CXCL16, DSC2, BIK, MUC16 |
| INTERPRO | **IPR020635:Tyrosine-protein kinase, catalytic domain** | 5 | 2,7 | **0,0082** | DDR1, FRK, PTK6, MST1R, EPHA1 |
| GOTERM_BP_DIRECT | **GO:0030335~positive regulation of cell migration** | 7 | 3,8 | **0,0087** | CXCL16, FAM110C, CEACAM6, LAMC2, FAM83H, EPHA1, GRB7 |
| GOTERM_CC_DIRECT | **GO:0005856~cytoskeleton** | 10 | 5,4 | **0,0095** | TNS4, JUP, MPZL2, EZR, EVPL, PLEK2, PPL, S100A9, MISP, FAM83H |
| GOTERM_BP_DIRECT | **GO:0032956~regulation of actin cytoskeleton organization** | 4 | 2,2 | **0,0103** | DIXDC1, EZR, ARHGEF5, GRHL3 |
| UP_KEYWORDS | **Amelogenesis imperfecta** | 3 | 1,6 | **0,0107** | LAMB3, ITGB6, FAM83H |
| GOTERM_CC_DIRECT | **GO:0016327~apicolateral plasma membrane** | 3 | 1,6 | **0,0112** | JUP, CLDN7, CLDN4 |
| GOTERM_MF_DIRECT | **GO:0004252~serine-type endopeptidase activity** | 8 | 4,3 | **0,0117** | KLK6, PRSS8, KLK8, ST14, PCSK9, KLK10, PRSS22, TMPRSS4 |
| UP_KEYWORDS | **Corneal dystrophy** | 3 | 1,6 | **0,0119** | OVOL2, TACSTD2, ZEB1 |
| INTERPRO | **IPR008266:Tyrosine-protein kinase, active site** | 5 | 2,7 | **0,0120** | DDR1, FRK, PTK6, MST1R, EPHA1 |
| KEGG_PATHWAY | **hsa04670:Leukocyte transendothelial migration** | 5 | 2,7 | **0,0124** | F11R, CLDN7, EZR, CLDN4, MAPK13 |
| UP_SEQ_FEATURE | **calcium-binding region:1; low affinity** | 3 | 1,6 | **0,0125** | S100P, S100A9, S100A14 |
| GOTERM_BP_DIRECT | **GO:0006970~response to osmotic stress** | 3 | 1,6 | **0,0126** | MAPK13, MARVELD3, MAP7 |
| SMART | **SM00219:TyrKc** | 5 | 2,7 | **0,0127** | DDR1, FRK, PTK6, MST1R, EPHA1 |
| GOTERM_CC_DIRECT | **GO:0048471~perinuclear region of cytoplasm** | 13 | 7,0 | **0,0162** | GALNT3, EPN3, ATP2C2, EZR, TSPAN1, LAMP3, MAL2, TMEM184A, PCSK9, CDH1, MAP7, LAMC2, S100A14 |
| UP_SEQ_FEATURE | **calcium-binding region:2; high affinity** | 3 | 1,6 | **0,0166** | S100P, S100A9, S100A14 |
| KEGG_PATHWAY | **hsa05412:Arrhythmogenic right ventricular cardiomyopathy (ARVC)** | 4 | 2,2 | **0,0169** | JUP, ITGB6, ITGB4, DSC2 |
| UP_KEYWORDS | **Tyrosine-protein kinase** | 5 | 2,7 | **0,0172** | DDR1, FRK, PTK6, MST1R, EPHA1 |
| UP_KEYWORDS | **Hypotrichosis** | 3 | 1,6 | **0,0172** | ST14, LIPH, CDH3 |
| UP_KEYWORDS | **Glycosyltransferase** | 7 | 3,8 | **0,0182** | GALNT3, ST3GAL2, B3GNT5, FUT3, B3GNT3, FUT2, ST6GALNAC1 |
| UP_SEQ_FEATURE | **compositionally biased region:Ser-rich** | 10 | 5,4 | **0,0189** | KIAA1522, TNS4, PLEKHG3, FAM83A, MUC20, CDH1, KRT4, TTC28, CDH3, MUC16 |
| GOTERM_CC_DIRECT | **GO:0005576~extracellular region** | 25 | 13,5 | **0,0191** | KLK6, ADGRF1, PLA2G10, IL18, S100A9, MUC20, SPINT1, CDCP1, CDH1, LCN2, PRSS8, LAMB3, ST3GAL2, PRRG4, LRG1, PRRG2, SPINT2, CXCL16, DMKN, LIPH, LAMC2, KLK10, PRSS22, FGFBP1, WFDC2 |
| INTERPRO | **IPR000233:Cadherin, cytoplasmic domain** | 3 | 1,6 | **0,0192** | DSC2, CDH1, CDH3 |
| OMIM_DISEASE | **226700~Epidermolysis bullosa, junctional, Herlitz type** | 2 | 1,1 | **0,0207** | LAMB3, LAMC2 |
| INTERPRO | **IPR000082:SEA domain** | 3 | 1,6 | **0,0208** | ADGRF1, ST14, MUC16 |
| UP_SEQ_FEATURE | **topological domain:Lumenal** | 10 | 5,4 | **0,0213** | GALNT3, LAMP3, ST3GAL2, B3GNT5, MAL2, VKORC1, FUT3, B3GNT3, FUT2, ST6GALNAC1 |
| GOTERM_MF_DIRECT | **GO:0008236~serine-type peptidase activity** | 4 | 2,2 | **0,0226** | PRSS8, ST14, KLK10, PRSS22 |
| UP_SEQ_FEATURE | **domain:MARVEL** | 3 | 1,6 | **0,0228** | MAL2, MARVELD2, MARVELD3 |
| KEGG_PATHWAY | **hsa04514:Cell adhesion molecules (CAMs)** | 5 | 2,7 | **0,0230** | F11R, CLDN7, CLDN4, CDH1, CDH3 |
| GOTERM_CC_DIRECT | **GO:0005882~intermediate filament** | 5 | 2,7 | **0,0231** | JUP, KRT19, KRT6A, KRT4, IFFO1 |
| KEGG_PATHWAY | **hsa00512:Mucin type O-Glycan biosynthesis** | 3 | 1,6 | **0,0233** | GALNT3, ST3GAL2, ST6GALNAC1 |
| INTERPRO | **IPR013761:Sterile alpha motif/pointed domain** | 5 | 2,7 | **0,0240** | CNKSR1, ELF3, EHF, ARAP2, EPHA1 |
| GOTERM_BP_DIRECT | **GO:0043087~regulation of GTPase activity** | 4 | 2,2 | **0,0245** | PROM2, ARHGEF5, EPHA1, ADAP1 |
| GOTERM_BP_DIRECT | **GO:0051384~response to glucocorticoid** | 4 | 2,2 | **0,0245** | IL1RN, PTPRU, SCGB1A1, ANXA3 |
| UP_KEYWORDS | **Phosphoprotein** | 87 | 47,0 | **0,0246** | CLDN7, CHMP4C, CLDN4, FAM110C, S100A9, FERMT1, CDCP1, TTC28, ZEB1, LSR, PAK6, FAM83B, KDF1, FAM83A, ATP8B2, FAM83H, C6ORF132, EPN3, F11R, LAD1, ARHGEF5, STAP2, ARHGEF16, ARHGAP27, PTPRU, TC2N, CDS1, GRHL1, SCEL, KIAA1522, TNS4, JUP, C19ORF33, DDR1, ATP2C2, KRT19, EVPL, SH2D3A, CGN, ESRP1, ESRP2, MST1R, MTFR1L, EPS8L1, FRK, PLEK2, TTC9, ITGB4, CDH1, KLC3, SFN, MACC1, LLGL2, ADAP1, PLEKHG3, EZR, PRRG4, OVOL2, DAPP1, MARVELD2, PPL, PTK6, PCSK9, DIXDC1, CNKSR1, TMEM45B, TRIM29, FOXA1, EXPH5, MISP, KCNK1, EPHA1, ANXA3, CBLC, PROM2, C1ORF116, MAPK13, PLEKHA7, PKP3, DSC2, MAP7, MYH14, TJP3, ARAP2, GRB7, TJP2, MUC16 |
| GOTERM_CC_DIRECT | **GO:0005911~cell-cell junction** | 6 | 3,2 | **0,0248** | JUP, F11R, CNKSR1, PKP3, PTPRU, GRHL2 |
| UP_KEYWORDS | **Phospholipase A2 inhibitor** | 2 | 1,1 | **0,0262** | SCGB1A1, ANXA3 |
| UP_KEYWORDS | **Cytoplasm** | 55 | 29,7 | **0,0270** | ELF3, CHMP4C, FAM110C, RNF39, S100A9, FERMT1, TTC28, PAK6, FAM83B, FAM83A, KDF1, FAM83H, ALS2CL, EPN3, ARHGEF5, STAP2, ARHGEF16, ARHGAP27, ELMO3, SCEL, JUP, TNS4, EVPL, TMEM184A, EPS8L1, FRK, PLEK2, MAL2, KLC3, SFN, MACC1, LLGL2, ADAP1, EZR, DAPP1, PTK6, PPL, PCSK9, DTX4, KLK6, DIXDC1, CNKSR1, KLK8, S100P, TRIM29, IL1RN, MISP, S100A14, C1ORF116, IRF6, PLEKHA7, SPRR1A, MAP7, ARAP2, GRB7 |
| INTERPRO | **IPR005417:Zona occludens protein** | 2 | 1,1 | **0,0277** | TJP3, TJP2 |
| GOTERM_CC_DIRECT | **GO:0044393~microspike** | 2 | 1,1 | **0,0284** | PROM2, EZR |
| GOTERM_BP_DIRECT | **GO:0090559~regulation of membrane permeability** | 2 | 1,1 | **0,0285** | F11R, TJP2 |
| GOTERM_BP_DIRECT | **GO:0010482~regulation of epidermal cell division** | 2 | 1,1 | **0,0285** | KDF1, SFN |
| INTERPRO | **IPR000219:Dbl homology (DH) domain** | 4 | 2,2 | **0,0287** | PLEKHG3, ARHGEF5, ARHGEF16, ALS2CL |
| KEGG_PATHWAY | **hsa04512:ECM-receptor interaction** | 4 | 2,2 | **0,0287** | LAMB3, ITGB6, ITGB4, LAMC2 |
| GOTERM_CC_DIRECT | **GO:0005887~integral component of plasma membrane** | 22 | 11,9 | **0,0289** | STEAP4, FXYD3, TSPAN1, CLDN4, TSPAN15, PTPRU, GPR87, KCNK1, EPHA1, EPCAM, DDR1, ATP2C2, PROM2, TACSTD2, PRRG2, ST14, CEACAM6, CEACAM5, B3GNT3, MST1R, SCNN1A, PTAFR |
| GOTERM_BP_DIRECT | **GO:0030855~epithelial cell differentiation** | 4 | 2,2 | **0,0297** | F11R, ELF3, EHF, KRT4 |
| INTERPRO | **IPR027397:Catenin binding domain** | 3 | 1,6 | **0,0297** | DSC2, CDH1, CDH3 |
| INTERPRO | **IPR013787:S100/CaBP-9k-type, calcium binding, subdomain** | 3 | 1,6 | **0,0297** | S100P, S100A9, S100A14 |
| INTERPRO | **IPR008253:Marvel** | 3 | 1,6 | **0,0297** | MAL2, MARVELD2, MARVELD3 |
| GOTERM_CC_DIRECT | **GO:0005938~cell cortex** | 5 | 2,7 | **0,0304** | CNKSR1, KDF1, FAM110C, LAMC2, MISP |
| UP_KEYWORDS | **Intermediate filament** | 4 | 2,2 | **0,0305** | KRT19, KRT6A, KRT4, IFFO1 |
| UP_KEYWORDS | **Palmoplantar keratoderma** | 3 | 1,6 | **0,0321** | JUP, KRT6A, GJB3 |
| UP_SEQ_FEATURE | **region of interest:Head** | 4 | 2,2 | **0,0325** | KRT19, KRT6A, CGN, KRT4 |
| GOTERM_CC_DIRECT | **GO:0009925~basal plasma membrane** | 3 | 1,6 | **0,0331** | CLDN4, TACSTD2, MUC20 |
| GOTERM_BP_DIRECT | **GO:0090004~positive regulation of establishment of protein localization to plasma membrane** | 3 | 1,6 | **0,0333** | EZR, ARHGEF16, AGR2 |
| GOTERM_CC_DIRECT | **GO:0005925~focal adhesion** | 9 | 4,9 | **0,0344** | TNS4, JUP, DIXDC1, EZR, ITGB6, FERMT1, CDH1, MISP, GRB7 |
| GOTERM_CC_DIRECT | **GO:0042995~cell projection** | 4 | 2,2 | **0,0350** | PROM2, EZR, ARHGEF5, GRB7 |
| SMART | **SM01394:SM01394** | 3 | 1,6 | **0,0354** | S100P, S100A9, S100A14 |
| GOTERM_MF_DIRECT | **GO:0005154~epidermal growth factor receptor binding** | 3 | 1,6 | **0,0355** | CBLC, FAM83B, AGR2 |
| GOTERM_BP_DIRECT | **GO:0008284~positive regulation of cell proliferation** | 10 | 5,4 | **0,0358** | KLF5, EPCAM, CLDN7, KRT6A, CEACAM6, RAB25, LAMC2, MST1R, FGFBP1, EPHA1 |
| GOTERM_CC_DIRECT | **GO:0032580~Golgi cisterna membrane** | 4 | 2,2 | **0,0362** | GALNT3, ST3GAL2, FUT3, FUT2 |
| INTERPRO | **IPR001664:Intermediate filament protein** | 4 | 2,2 | **0,0364** | KRT19, KRT6A, KRT4, IFFO1 |
| GOTERM_CC_DIRECT | **GO:0061689~tricellular tight junction** | 2 | 1,1 | **0,0377** | MARVELD2, LSR |
| GOTERM_BP_DIRECT | **GO:0002159~desmosome assembly** | 2 | 1,1 | **0,0378** | JUP, PKP3 |
| GOTERM_MF_DIRECT | **GO:0019834~phospholipase A2 inhibitor activity** | 2 | 1,1 | **0,0378** | SCGB1A1, ANXA3 |
| GOTERM_MF_DIRECT | **GO:0030674~protein binding, bridging** | 4 | 2,2 | **0,0379** | CNKSR1, EVPL, SPRR1A, TJP2 |
| UP_KEYWORDS | **Non-syndromic deafness** | 4 | 2,2 | **0,0414** | MARVELD2, GJB3, MYH14, GRHL2 |
| UP_SEQ_FEATURE | **site:Stutter** | 3 | 1,6 | **0,0417** | KRT19, KRT6A, KRT4 |
| INTERPRO | **IPR001245:Serine-threonine/tyrosine-protein kinase catalytic domain** | 5 | 2,7 | **0,0422** | DDR1, FRK, PTK6, MST1R, EPHA1 |
| GOTERM_MF_DIRECT | **GO:0005509~calcium ion binding** | 13 | 7,0 | **0,0436** | GALNT3, CBLC, S100P, PRRG4, PLA2G10, PRRG2, S100A9, DSC2, CDH1, TC2N, CDH3, S100A14, ANXA3 |
| UP_SEQ_FEATURE | **domain:PH 2** | 3 | 1,6 | **0,0439** | PLEK2, ARAP2, ADAP1 |
| GOTERM_BP_DIRECT | **GO:0008543~fibroblast growth factor receptor signaling pathway** | 4 | 2,2 | **0,0442** | GALNT3, ESRP1, ESRP2, FGFBP1 |
| SMART | **SM01391:SM01391** | 4 | 2,2 | **0,0456** | KRT19, KRT6A, KRT4, IFFO1 |
| GOTERM_BP_DIRECT | **GO:0043547~positive regulation of GTPase activity** | 11 | 5,9 | **0,0458** | F11R, PLEKHG3, SH2D3A, ARHGEF5, ARHGAP27, GRHL3, ARAP2, EPS8L1, LLGL2, ADAP1, ALS2CL |
| UP_KEYWORDS | **Cleavage on pair of basic residues** | 7 | 3,8 | **0,0460** | KLK6, PRRG4, PLA2G10, DSC2, CDH1, MST1R, CDH3 |
| UP_SEQ_FEATURE | **domain:PH 1** | 3 | 1,6 | **0,0460** | PLEK2, ARAP2, ADAP1 |
| UP_KEYWORDS | **Keratinization** | 3 | 1,6 | **0,0461** | EVPL, SPRR1A, PPL |
| GOTERM_BP_DIRECT | **GO:0071681~cellular response to indole-3-methanol** | 2 | 1,1 | **0,0470** | JUP, CDH1 |
| GOTERM_BP_DIRECT | **GO:0031346~positive regulation of cell projection organization** | 2 | 1,1 | **0,0470** | LCN2, PROM2 |
| UP_KEYWORDS | **Basement membrane** | 3 | 1,6 | **0,0482** | LAMB3, LAD1, LAMC2 |
| UP_KEYWORDS | **Signal-anchor** | 9 | 4,9 | **0,0485** | GALNT3, ST3GAL2, B3GNT5, ST14, FUT3, B3GNT3, FUT2, TMPRSS4, ST6GALNAC1 |
| GOTERM_BP_DIRECT | **GO:0007166~cell surface receptor signaling pathway** | 7 | 3,8 | **0,0487** | CBLC, ADGRF1, TSPAN1, TACSTD2, TSPAN15, EPHA1, ADAP1 |
| GOTERM_BP_DIRECT | **GO:0034332~adherens junction organization** | 3 | 1,6 | **0,0489** | JUP, CDH1, CDH3 |
