# Supplementary material for: Integrative analysis of the cancer genome atlas and cancer cell lines encyclopedia large-scale genomic databases: MUC4/MUC16/MUC20 signature is associated with poor survival in human carcinomas
Source: J Transl Med. 2018 Sep 20;16:259. doi: 10.1186/s12967-018-1632-2 (PMC6149062; doi:10.1186/s12967-018-1632-2)
Supplement: Supplementary file 6 — Additional file 6: Table S2. Hazard-ratio and survival analysis of most significant genes clustered in GO term associated with MUC4 expression in TCGA tumor databases. Hazard ratio and p-value were determined using SurvExpress tool (http://bioinformatica.mty.itesm.mx/SurvExpress). Risk groups were sorted depending on the major GO term GO 0031424, GO 00071555, GO 0019897, GO 0016323 and GO 0016324 using the optimization algorithm (maximize) from the ordered prognostic. [file 12967_2018_1632_MOESM6_ESM.docx]

Additional file 6: Table S2 : **Hazard-ratio and survival analysis of most significant genes clustered in GO term associated with MUC4 expression in TCGA tumor databases.** Hazard ratio and p-value were determined using SurvExpress tool (<http://bioinformatica.mty.itesm.mx/SurvExpress>). Risk groups were sorted depending on the major GO term GO 0031424, GO 00071555, GO 0019897, GO 0016323 and GO 0016324 using the optimization algorithm (maximize) from the ordered prognostic.

| **GO term** | **genes** | **TCGA dataset** | **Hazard ratio [95% CI]** | **Log rank equal curves** | **P value** |
| --- | --- | --- | --- | --- | --- |
| **GO:0031424 keratinization** | EVPL, SPRR1A, PPL, SFN, CDH3 | Bladder BLCA | 2.74 [2.02 ; 3.72] | p=1.862e−11 | p=1.144e−10 |
|  |  | Colon COADREAD | 2.15 [1.38 ; 3.36] | p=0.0005669 | p=0.0007724 |
|  |  | Lung ADK LUAD | 1.65 [1.17 ; 2.33] | p=0.004055 | p=0.004455 |
|  |  | Lung Squamous LUSC | 2.55 [1.6 ; 4.06] | p=4.574e−05 | p=7.959e−05 |
|  |  | Ovarian serous cystadenoma | 1.85 [1.23 ; 2.77] | p=0.002727 | p=0.00319 |
|  |  | Pancreatic PAAD | 2.28 [1.44 ; 3.59] | p=0.0002917 | p=0.0004229 |
|  |  | Skin SKCM | 3.76 [2.39 ; 5.89] | p=6.875e−10 | p=8.632e−09 |
|  |  | Stomach STAD | 1.67 [1.15 ; 2.43] | p=0.006136 | p=0.00674 |
| **GO:0007155 cell adhesion** | JUP, DDR1, F11R, LAMB3, ITGB6, ITGB4, FERMT1, DSC2, LAMC2, PTPRU, CDH3, GRHL2, MUC16 | Bladder BLCA | 2.89 [2.06 ; 4.07 | p=1.506e−10 | p=9.605e−10 |
|  |  | Colon COADREAD | 2.47 [1.64 ; 3.71] | p=7.798e−06 | p=1.555e−05 |
|  |  | Lung ADK LUAD | 2.46 [1.64 ; 3.69] | p=6.7e−06 | p=1.328e−05 |
|  |  | Lung Squamous LUSC | 2.68 [1.58 ; 4.55] | p=0.0001459 | p=0.0002616 |
|  |  | Ovarian serous cystadenoma | 2.76 [1.89 ; 4.03] | p=4.157e−08 | p=1.429e−07 |
|  |  | Pancreatic PAAD | 3.23 [2.06 ; 5.07] | p=7.162e−08 | p=3.405e−07 |
|  |  | Skin SKCM | 3 [2.1 ; 4.29] | p=2.612e−10 | p=1.682e−09 |
|  |  | Stomach STAD | 2.15 [1.45 ; 3.19] | p=9.644e−05 | p=0.0001411 |
| **GO:0019897 extrinsic component of plasma membrane** | PRSS8, EPN3, ST14, PRSS22 | Bladder BLCA | 2.36 [1.5 ; 3.73] | p=0.0001433 | p=0.0002271 |
|  |  | Colon COADREAD | 2.06 [1.35 ; 3.13] | p=0.0005707 | p=0.0007519 |
|  |  | Lung ADK LUAD | 1.66 [1.11 ; 2.5] | p=0.01326 | p=0.01429 |
|  |  | Lung Squamous LUSC | 2.34 [1.49 ; 3.69] | p=0.0001563 | p=0.0002391 |
|  |  | Ovarian serous cystadenoma | 1.55 [1.11 ; 2.17] | p=0.009375 | p=0.009998 |
|  |  | Pancreatic PAAD | 2.5 [1.08 ; 5.79] | p=0.02667 | p=0.03194 |
|  |  | Skin SKCM | 1.34 [0.99 ; 1.8] | ns  p=0.05927 | ns  p=0.06018 |
|  |  | Stomach STAD | 1.84 [1.27 ; 2.68] | p=0.001042 | p=0.001246 |
| **GO:0016323 basolateral plasma membrane** | EPCAM, CLDN7, PROM2, EZR, MARVELD2, ST14, MAP7, CEACAM5 | Bladder BLCA | 2.21 [1.48 ; 3.3] | p=7.429e−05 | p=0.0001136 |
|  |  | Colon COADREAD | 2.24 [1.47 ; 3.41] | p=0.0001125 | p=0.0001688 |
|  |  | Lung ADK LUAD | 1.52 [0.98 ; 2.37] | ns  p=0.05931 | ns  p=0.06122 |
|  |  | Lung Squamous LUSC | 2.34 [1.35 ; 4.07] | p=0.001827 | p=0.002487 |
|  |  | Ovarian serous cystadenoma | 2.36 [1.43 ; 3.88] | p=0.0004957 | p=0.0007228 |
|  |  | Pancreatic PAAD | 4.5 [2.06 ; 9.81] | p=3.936e−05 | p=0.0001593 |
|  |  | Skin SKCM | 2.63 [1.75 ; 3.95] | p=1.423e−06 | p=3.441e−06 |
|  |  | Stomach STAD | 2.39 [1.69 ; 3.37] | p=3.572e−07 | p=8.04e−07 |
| **GO:0016324 apical plasma membrane** | EPCAM, PROM2, EZR, CLDN4, MAL2, MARVELD2, MUC20, CRB3, KCNK1, SCNN1A | Bladder BLCA | 3.13 [2.14 ; 4.59] | p=5.886e−10 | p=4.3e−09 |
|  |  | Colon COADREAD | 2.07 [1.38 ; 3.11] | p=0.0003139 | p=0.0004242 |
|  |  | Lung ADK LUAD | 1.71 [1.25 ; 2.32] | p=0.0005921 | p=0.0006846 |
|  |  | Lung Squamous LUSC | 2.43 [1.54 ; 3.83] | p=8.513e−05 | p=0.000137 |
|  |  | Ovarian serous cystadenoma | 1.8 [1.28 ; 2.54] | p=0.0005943 | p=0.0007114 |
|  |  | Pancreatic PAAD | 4.42 [2.04 ; 9.61] | p=4.184e−05 | p=0.0001722 |
|  |  | Skin SKCM | 2.56 [1.59 ; 4.12] | p=6.298e−05 | p=0.0001136 |
|  |  | Stomach STAD | 2.48 [1.69 ; 3.64] | p=1.821e−06 | p=3.949e−06 |
