# Supplementary material for: Integrative analysis of the cancer genome atlas and cancer cell lines encyclopedia large-scale genomic databases: MUC4/MUC16/MUC20 signature is associated with poor survival in human carcinomas
Source: J Transl Med. 2018 Sep 20;16:259. doi: 10.1186/s12967-018-1632-2 (PMC6149062; doi:10.1186/s12967-018-1632-2)
Supplement: Supplementary file 7 — Additional file 7: Table S3. Hazard-ratio and survival analysis of top genes associated with MUC4 expression in TCGA tumor databases. Hazard ratio and p-value were determined using SurvExpress tool (http://bioinformatica.mty.itesm.mx/SurvExpress). Risk groups were defined using the optimization algorithm (maximize) from the ordered prognostic. Selected genes (ADGRF1, LCN2, MUC20, C1ORF116, SCEL, STEAP4) harbored Pearson’s correlation with MUC4 > 0.5. [file 12967_2018_1632_MOESM7_ESM.docx]

Additional file 7: Table S3 : **Hazard-ratio and survival analysis of top genes associated with MUC4 expression in TCGA tumor databases.** Hazard ratio and p-value were determined using SurvExpress tool (<http://bioinformatica.mty.itesm.mx/SurvExpress>). Risk groups were defined using the optimization algorithm (maximize) from the ordered prognostic. Selected genes (ADGRF1, LCN2, MUC20, C1ORF116, SCEL, STEAP4) harbored Pearson’s correlation with MUC4 > 0.5.

| **Genes** | **TCGA dataset** | **Hazard ratio [95% CI]** | **Log rank equal curves** | **P value** |
| --- | --- | --- | --- | --- |
| ADGRF1  LCN2  C1ORF116  SCEL  STEAP4  MUC20 | Bladder BLCA | 2.51 [1.83 ; 3.45] | p=3.846e−09 | p=1.28e−08 |
|  | Colon COADREAD | 2.22 [1.41 ; 3.49] | p=0.0004004 | p=0.0005655 |
|  | Lung ADK LUAD | 1.91 [1.41 ; 2.6] | p=2.318e−05 | p=3.191e−05 |
|  | Lung Squamous LUSC | 2.01 [1.17 ; 3.45] | p=0.01044 | p=0.01189 |
|  | Ovarian serous cystadenoma | 2.14 [1.37 ; 3.34] | p=0.000599 | p=0.0008063 |
|  | Pancreatic PAAD | 8.77 [2.15 ; 35.83] | p=0.0002847 | p=0.002505 |
|  | Skin SKCM | 2.01 [1.42 ; 2.84] | p=6.132e−05 | p=8.554e−05 |
|  | Stomach STAD | 2.19 [1.51 ; 3.18] | p=2.489e−05 | p=3.945e−05 |
